# Theory Guided Fine‐Tune of Strain Effects in Pt Ternary Alloy via Rare Earth Templating: Achieving High Performance PEMFCs Catalysts

**DOI:** 10.1002/adma.73269

**Published:** 2026-05-18

**Authors:** Qi Zhang, Hong Zhang, Sungho Jeon, Erika Ortega Ortiz, Brooke E. Vander Pas, Guangqi Zhu, Yi‐Kai Lien, Chenzhao Li, Huayu Guo, Baixu Zhu, Yaroslav Losovyj, Gabriel M. Filippelli, Xingchen Ye, Eric A Stach, Ping Liu, Jian Xie

**Affiliations:** ^1^ School of Mechanical Engineering Purdue University West Lafayette Indiana USA; ^2^ Department of Chemistry Stony Brook University Stony Brook New York USA; ^3^ Department of Materials Science and Engineering University of Pennsylvania Philadelphia Pennsylvania USA; ^4^ Department of Earth and Environmental Sciences Indiana University Indianapolis Indiana USA; ^5^ School of Materials Engineering Purdue University West Lafayette Indiana USA; ^6^ Department of Chemistry Indiana University Bloomington Indiana USA; ^7^ Chemistry Division Brookhaven National Laboratory Upton New York USA

**Keywords:** ORR, PEMFCs, Pt ternary alloys, rare earth metal, strain effects, theory‐guided design, volcano plots

## Abstract

The sluggish kinetics and insufficient durability of platinum‐based catalysts remain crucial barriers limiting proton‐exchange‐membrane fuel cells (PEMFCs) deployment. Here, we report a theory‐guided synthesis combined with rare‐earth templating to realize a previously inaccessible Pt_5_Co‐like phase with tailored atomic‐scale strain. Guided by density functional theory (DFT) calculations, we identified that a Pt_5_Co‐like sublayer can induce a unique mild compressive strain (−1.24%) to the Pt(111) shell and an optimal *OH binding energy shift (Δ*E* ≈ 0.11 *eV*). This shift positions the alloy catalyst near the apex of the oxygen reduction reaction activity volcano. This prediction guided the synthesis of ternary alloy Pt_5_(Ce)Co@Pt multilayer nanoparticles, featuring a Ce‐stabilized core, a Pt_5_Co‐like sublayer, and a Pt‐rich shell. This catalyst demonstrates both exceptionally high activity and durability, achieving a mass activity of 2.6 A∙mg_Pt_^−1^ in rotating disk electrode testing. In fuel cell membrane electrode assembly tests, Pt_5_(Ce)Co@Pt achieves a current density of 1.9 A∙cm^−2^ at 0.7 V under heavy‐duty vehicle conditions. Remarkably, it maintains 1.2 A∙cm^−2^ after 1 80 000 AST cycles, doubling the U.S. DOE 2025 target. This work demonstrates a rational design strategy that DFT‐guided strain engineering integrates with rare‐earth templating to advance Pt‐based catalysts for fuel cell applications.

## Graphical abstract

Theory‐guided strain engineering and rare‐earth templating are combined to create Pt_5_(Ce)Co@Pt multilayer nanoparticles with a Pt_5_Co‐like sublayer that fine‐tunes *OH binding near the ORR volcano optimum. The resulting catalyst delivers exceptional mass activity and remarkable long‐term PEMFC durability, revealing a strategy for designing highly active and stable Pt‐based fuel‐cell electrocatalysts.

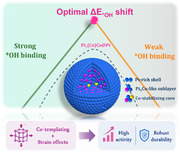

## 1 Introduction

The oxygen reduction reaction (ORR) at the cathode of proton exchange membrane fuel cells (PEMFCs) represents a critical performance bottleneck due to its slow reaction kinetics. Platinum (Pt)‐based catalysts remain a benchmark for ORR activity, yet their high cost, limited availability, and performance degradation under operational conditions pose significant obstacles to widespread commercial adoption [1, 2]. The sluggish kinetics of ORR on stable Pt(111) surfaces have been attributed to the over‐binding of oxygen intermediate species, such as *O or *OH, which impedes their removal during H_2_O formation, causing a substantial potential drop in the ORR process [3, 4, 5]. One established approach to enhance ORR activity is to alloy Pt with late transition metals (Pt–M, where M = Cobalt (Co), Nickel (Ni), Iron (Fe), etc.), which has been shown to simultaneously improve the intrinsic activity and reduce the Pt content [6, 7, 8, 9, 10, 11]. These Pt‐M alloys introduce compressive lattice strain and electronic (ligand) effects, which can weaken the Pt‐OH bond strength and shift the catalyst closer to the apex of the ORR volcano plot [4, 12]. Among them, Pt‐Co alloys have been extensively studied, with Pt_3_Co and PtCo intermetallics showing promising activity improvement [13, 14, 15]. However, these alloys often suffer from structural instability and excessive lattice compression that shifts the catalyst performance beyond the optimal *OH binding point, especially under fuel cell operation, leading to reduced catalytic activity [4, 12, 16]. Moreover, conventional synthesis approaches provide limited control over atomic‐scale arrangements and surface strain engineering, hindering precise catalyst optimization.

As studied through density functional theory (DFT) calculations by Nørskov and co‐workers, a key relationship between ORR activity of Pt alloys and *OH binding energy is established [12, 17]. This is commonly described by what are called “volcano plots”, due to their shape. Peak performance occurs when *OH binding is 0.10–0.12 eV weaker than on pure Pt(111) [5, 12]. This insight has guided the design of Pt‐based alloys for over a decade. Researchers have sought design strategies that fine‐tune the Pt‐OH interaction to achieve optimal binding energy and maximize ORR activity. However, this fine‐tuning requires precise measurement of how alloying changes binding properties. This measurement is difficult because Pt alloy nanoparticles have limited structural control under ORR conditions, making it hard to quantify changes in shell structures and binding properties.

Recent studies have shown that alloying Pt with rare earth (RE) metals can significantly enhance structural and thermodynamic stability, and provide several unique advantages for ORR catalyst design beyond those achievable with conventional Pt‐M alloys [18, 19, 20]. First, Pt‐RE alloys generally possess highly negative formation enthalpies, which strengthen metal‐metal bonding and suppress surface segregation, dissolution, and structural collapse under electrochemical operation [21, 22, 23]. This thermodynamic stabilization is particularly attractive for improving the long‐term durability of Pt‐based catalysts. RE species can also perturb the electronic structure of host materials through orbital interactions involving RE *4f/5d* states and neighboring ligand or metal states [24, 25, 26]. Such RE‐induced electronic modulation has recently been discussed in terms of gradient orbital coupling, where *f‐p‐d* interactions can tune the adsorption properties of catalytic surfaces and optimize reaction energetics. These RE‐induced modulations were distinguished in energy electrocatalysis materials, including oxygen evolution reaction, hydrogen evolution reaction, carbon dioxide reduction reaction, and ORR [19, 25, 27, 28]. Second, RE elements provide an effective route for fine‐tuning lattice strain [26, 29, 30, 31]. Pt‐RE alloys and some rare earth oxides enable precise lattice strain engineering through the lanthanide contraction effect. As the covalent radii of RE elements decrease across the lanthanide series, the lattice constant of the resulting Pt‐RE alloys contracts, leading to shorter Pt‐Pt bond distances and introducing compressive strain into the Pt lattice [31, 32]. This induced compressive strain can be used to systematically modulate the binding strength of key oxygen intermediates such as *OH, positioning the surface reactivity closer to the volcano plot optimum [5]. Third, Pt–RE systems often form unusual intermetallic structures differing from Pt‐M intermetallic alloys, such as CaCu_5_‐type hexagonal phases [18, 19]. These crystallographic motifs are distinct from traditional face‐centered cubic (fcc) or body‐centered cubic (bcc) structures, and can offer a platform for integrating additional elements and stabilizing otherwise inaccessible local atomic arrangements. Therefore, introducing RE elements into Pt‐based alloys is not only beneficial for improving stability, but also offers a way to direct phase evolution and create strain‐engineered architectures. Herein, Cerium (Ce) was selected because it can simultaneously stabilize the Pt_5_Ce CaCu_5_‐type hexagonal framework and template the formation of a Pt_5_Co‐like structure. Integrating lanthanide structural ordering with transition metals like Co represents a promising pathway to developing Pt‐RE‐M ternary alloys that simultaneously optimize catalytic activity and surface stability.

In this work, we develop a design strategy to precisely control the amount of Co in a Pt‐Co intermetallic layer to achieve the optimal strain imposed on the Pt shell (Pt_x_Co@Pt) and, consequently, optimize ORR performance. The strategy combines the thermodynamic and structural stability of Pt‐Ce intermetallics with the activity‐enhancing strain effects of Pt‐Co alloys. Specifically, the DFT calculations quantified a critical compressive strain on the order of ∼1.0%–1.4% that is required to weaken *OH binding to the optimal amount (ΔE_*OH_ ≈ 0.10–0.12 eV weaker than Pt(111)) and thus yield optimal ORR activity, as previously proposed [5, 12]. It is shown that Pt_x_Co@Pt core–shell structures can achieve this compressive strain while improving both ORR activity and stability compared to pure Pt [3, 33, 34]. By decreasing the Co content from PtCo (1:1 Pt:Co), to Pt_3_Co (3:1 Pt:Co) to Pt_5_Co (5:1 Pt:Co) via a Pt_x_Co(111)@Pt slab model, the DFT study discovers that Pt_5_Co (111) sublayers are able to induce about −1.24% lattice strain on the Pt(111) shell, falling within the target range. Bulk Pt_5_Co is not thermodynamically stable, but we overcame this by stabilizing it via Ce‐templating. Herein, we developed a synthesis route to produce ternary alloy of Pt_5_(Ce)Co@Pt multilayered nanoparticles, comprising a Ce‐rich stabilizing core, a Pt_5_Co‐like sublayer, and a protective Pt shell. Indeed, the DFT‐calculated *OH binding on the Pt(111) shell correlated well with the experimentally measured ORR activity, following a volcano trend. Pt_5_(Ce)Co@Pt displays a superior ORR activity to Pt_3_Co@Pt, PtCo@Pt, and commercial Pt/C in both rotating disk electrode (RDE) and membrane electrode assembly (MEA) measurements. Remarkably, the catalyst not only achieves U.S. Department of Energy (DOE) 2025 activity targets but also exceeds DOE heavy‐duty‐vehicle (HDV) durability benchmarks (maintaining 1.07 A∙cm^−2^ at 0.7 V after 90 000 AST cycles) even after 1 80 000 AST cycles, doubling the DOE target. Through this integrated theoretical and experimental approach, our study highlights the crucial quantification of alloying effect on the structure and binding properties to enable the fine‐tuning of ORR activity near the optimal region while maintaining robust activity, facilitating the design of next‐generation Pt‐based catalysts for large‐scale application of PEMFCs.

## 2 Results and Discussion

### 2.1 DFT Calculations and Volcano Plots Guided Synthesis

**FIGURE 1:**
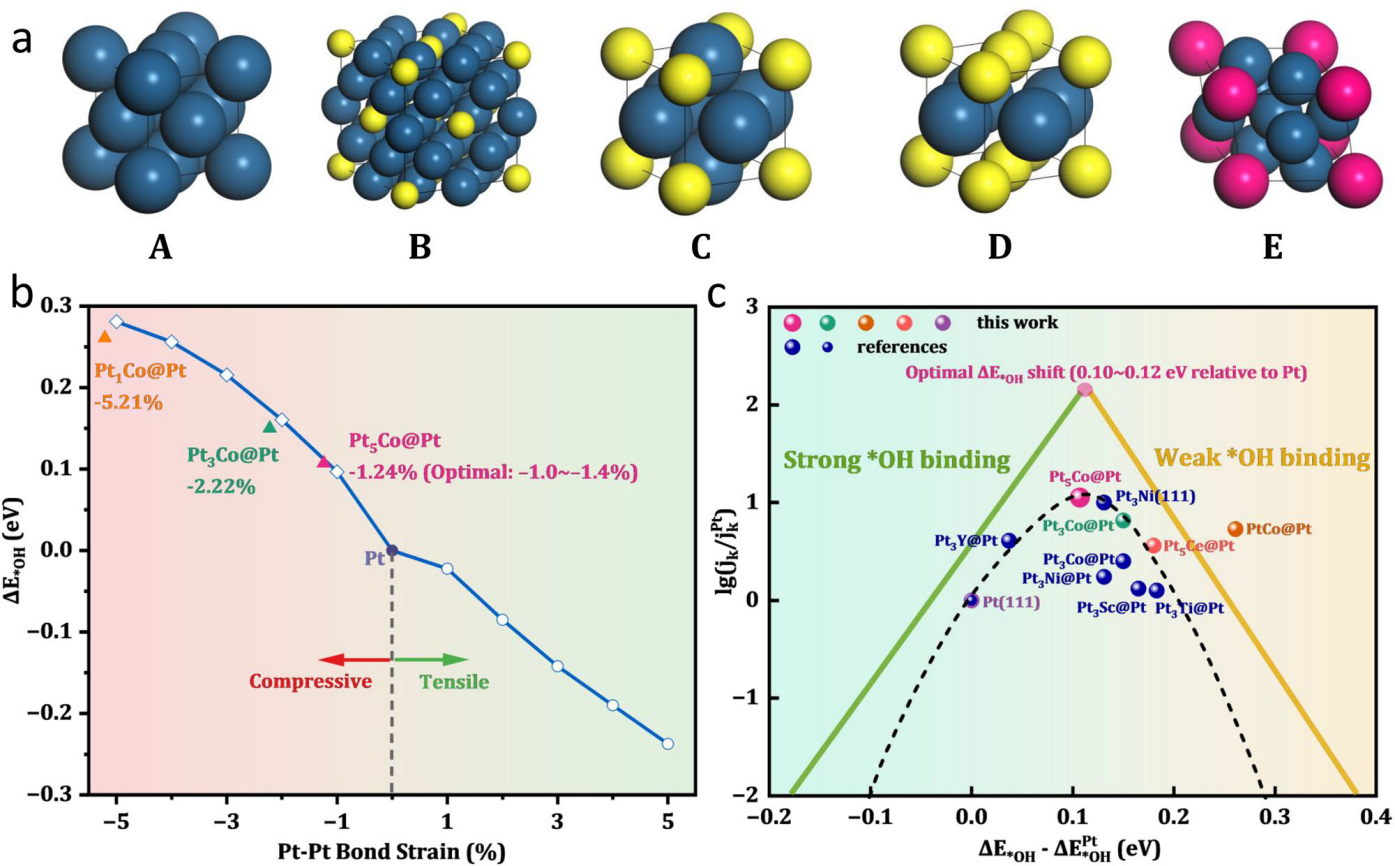
(a) Bulk lattice of (A) Pt, (B) Pt_5_Co (Pt_27_Co_5_), (C) Pt_3_Co, (D) Pt_1_Co, and (E) Pt_5_Ce (Blue: Pt, Yellow: Co, Pink: Ce). (b) DFT calculated Pt‐Pt bond strain on Pt_x_Co@Pt vs. ΔE_*OH_ respect to Pt(111); (c) ΔE_*OH_ vs. mass activity volcano plot (Volcano model is adapted from Greeley [17] and Norskov et al. [12]., reference datapoints marked by royal blue are from literatures: Pt(111) [37], Pt_3_Y@Pt [17], Pt_3_Ni(111) [5], Pt_3_Sc@Pt [38], Pt_3_Ni@Pt, Pt_3_Co@Pt, Pt_3_Ti@Pt [39]. Copyright 2018 American Chemical Society.

According to our recent studies of M@Pt core–shell nanostructures, the strain effects imposed by the core on the shell dominate the tuning of binding properties and ORR activities, while electronic interactions through charge transfer from core to shell are rather subtle [14]. We initiated this study by performing DFT calculations to quantify the relationship between lattice strain and *OH binding energetics on Pt. The slab models showed a linear decrease in *OH binding energy with an increasing compressive strain in the lattice (Figure 1a,b), consistent with previous calculations [35]. Recent works show that optimal ORR activity occurs when *OH binding is 0.10c−0.12 eV weaker than on pure Pt, corresponding to −1.0% to −1.4% compressive strain (Figure 1c) [5, 14, 36]. To achieve this optimal strain, we constructed slab models with Pt_x_Co(111) cores covered by three Pt(111) shell layers (Figure S1). We varied the Pt:Co ratio in the core from Pt_5_Co(111) to Pt_3_Co(111) to Pt_1_Co(111) (Figure 1a). Decreasing the Pt:Co ratio increases the Co content, which enlarges the core lattice constant and increases compressive strain on the Pt shell, as marked in Figure 1b.

The DFT calculations confirm our predicted strain‐ΔE_*OH_ correlation (Figure 1c). Pt_5_Co(111)@Pt produces optimal *OH binding (ΔE*OH = 0.11 eV) with minimal surface strain (−1.24%), falling within the target range of 0.10–0.12 eV for maximum ORR activity (Table 1). Higher Co content creates excessive strain that weakens *OH binding too much. Pt_3_Co(111)@Pt shows −2.22% strain, while PtCo(111)@Pt shows −5.21% strain. This increased strain shifts the Pt *d*‐band center downward and weakens *OH binding to 0.15 and 0.26 eV, respectively, both beyond the optimal range (Table 1). These results demonstrate that strain effects dominate *OH binding in these core–shell structures. By comparison, the contributions from ligand and ensemble effects are negligible. Controlled calculations where we fixed the Pt(111) shell strain while varying the Pt_x_Co core composition showed no change in ΔE*_OH_, confirming no ligand effect due to the limited charge transfer through the three‐layer Pt(111) shell (Table S1). Besides, both Pt_5_Co‐like sub‐layer and Pt_5_Ce inner core do not participate in the reaction directly, excluding the ensemble effect.

**TABLE 1:** DFT calculated Pt_x_Co@Pt properties including strain, d‐band center, and relative binding energy of *OH of surface Pt atoms with respect to Pt(111).

Composition	Pt‐Pt surface strain (%)	Pt d‐band center (ε_d_, eV)	ΔE_*OH_ (eV)
Pt(111)	0.00	0.00	0.00
Pt_5_Co@Pt	−1.24	−0.02	0.107
Pt_3_Co@Pt	−2.22	−0.24	0.150
Pt_1_Co@Pt	−5.21	−0.55	0.261

Guided by the DFT predictions, we synthesized core–shell (or, sublayer‐shell) nanoparticles with varying compositions: Pt_5_(Ce)Co@Pt, Pt_3_Co@Pt, and PtCo@Pt. Particle sizes were kept constant and sufficiently large (mean ≈ 5 nm) to minimize size effects, enabling fair comparison and validation of the slab model predictions (Figure S3). Based on the DFT calculated phase diagram (Figure S4), PtCo is the most stable intermetallic phase (formation energy: *E*_f_ = −0.09 eV/atom), followed by Pt_3_Co (*E*_f_ = −0.07 eV) and Pt_5_Co (*E*_f_ = −0.03 eV/atom) in a decreasing sequence. This implies Pt_5_Co is not a thermodynamically favored bulk phase. To overcome this limitation, we created a Pt_5_Co‐like architecture by synthesizing a Pt_5_Ce precursor and then replacing Ce with Co using a Co‐favorable high‐temperature anneal in diluted H_2_ (Figure S5). The material was subjected to acid leaching to remove surface oxides and segregated Ce, and to form a Pt‐rich shell, yielding the desired Pt_5_(Ce)Co@Pt multilayer structure (details see Supporting Information on Experiments Section). Inductively coupled plasma atomic emission spectroscopy (ICP‐AES) showed that the binary Pt_5_Ce precursor had a Pt:Ce atomic ratio around 5:1, while the final post‐leaching ternary Pt_5_(Ce)Co@Pt material had a Pt:Co ratio of approximately 7:1, with only ∼3.4 at.% Ce remaining. The compositional analysis confirms that Co diffused into the Pt_5_Ce lattice, replacing most of the Ce atoms and creating a Pt‐Co intermetallic phase in the particle sublayer. The following acid leaching process removed excessive Co in the near‐surface region and formed a Pt‐rich shell, yielding a Pt:Co ratio less than 5:1.

To further clarify the role of Ce in enabling this metastable architecture, we performed additional DFT calculations to evaluate the substitution energetics of Ce by Co in both bulk Pt_5_Ce and slab Pt_5_Ce@Pt models. The substitution of Ce by Co in bulk Pt_5_Ce was found to be energetically viable (Δ*E* = −1.30 eV/atom); however, this process requires a structural transformation from the *CaCu_5_‐type* hexagonal lattice Pt_5_Ce to the *fcc* lattice Pt_5_Co (Figure 1a), which is expected to be kinetically hindered under the experimental conditions. As a result, the Ce in the inner core is largely preserved. In contrast, when Ce atoms located at the subsurface of a Pt(111)/Pt_5_Ce slab were replaced by Co, the substitution became more energetically favorable (Δ*E* = −2.58 eV/atom, Figure S2). This behavior suggests that Co incorporation is not only thermodynamically driven, but also site‐dependent in the subsurface region. Consequently, Co preferentially replaces Ce in the subsurface region, leading to the formation of a Pt_5_Co‐like sublayer beneath the Pt shell, while Pt_5_Ce is retained in the inner core due to both kinetic limitations and its intrinsic thermodynamic stability. Herein, the DFT calculations describe well the experimentally observed multilayer structure consisting of a Ce‐rich core, a Pt_5_Co‐like sublayer, and a Pt‐rich shell, that is, the Ce atom acts as a structural and thermodynamic template that enables formation of a metastable Pt_5_Co‐like sublayer, which in turn imposes the optimal compressive strain (∼−1.24%) and thereby ORR activity on the Pt shell.

### 2.2 Structural Characterizations

**FIGURE 2:**
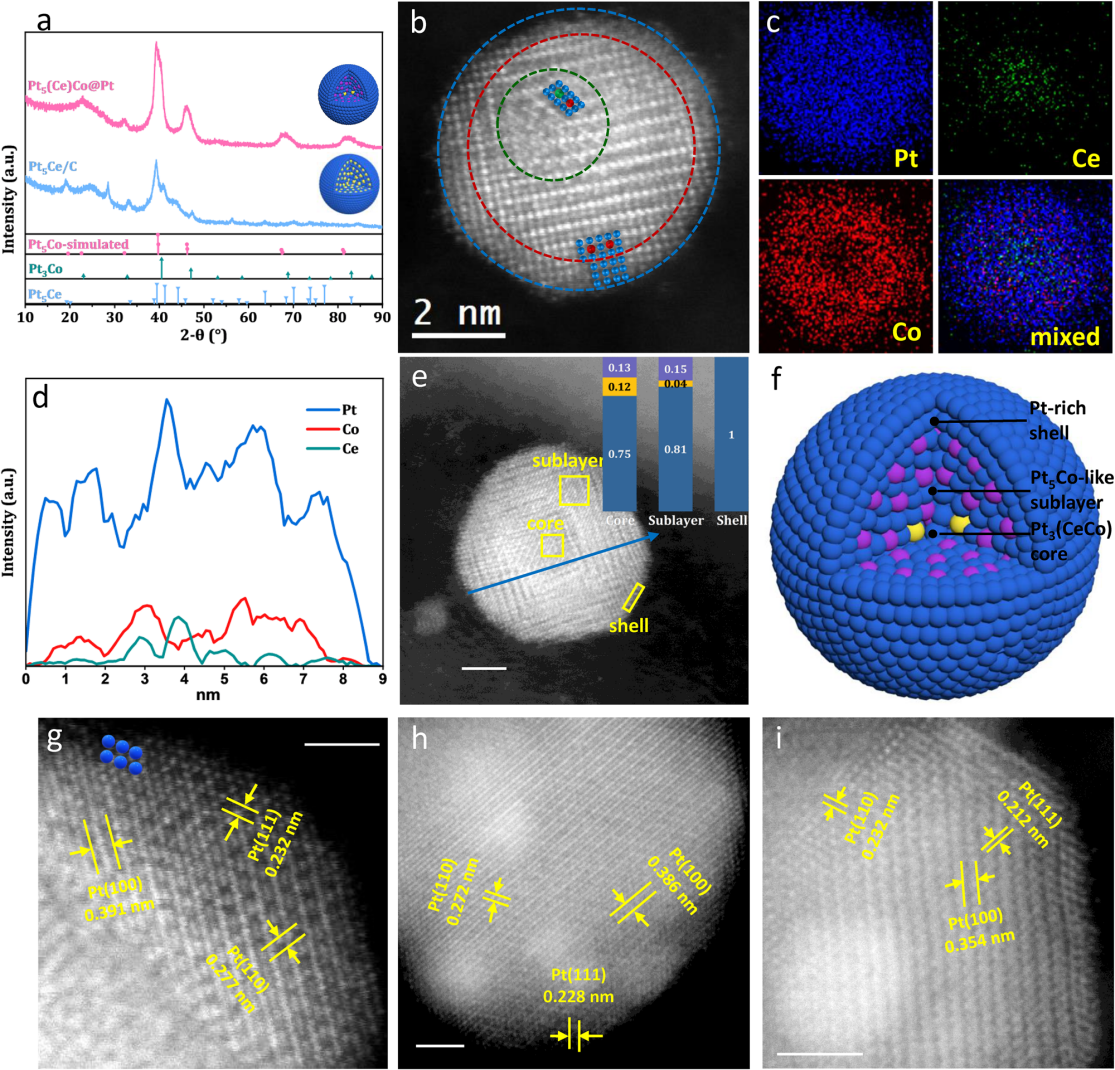
(a) XRD patterns of binary precursor Pt_5_Ce supported on Ketjen carbon black (in blue) and Pt_5_(Ce)Co@Pt (in pink); (b) HAADF‐STEM image of representative nanoparticle and (c) EDS mapping and (d) Line scan profiles of Pt_5_(Ce)Co@Pt; (e) Differential area mapping of Pt_5_(Ce)Co@Pt, inserted bar chart illustrates the elemental distribution at each selected area; (f) Schematic of Pt_5_(Ce)Co@Pt nanoparticle; HAADF‐STEM images of (g) Pt_5_(Ce)Co@Pt particle, (h) Pt_3_Co@Pt particle and (i) PtCo@Pt particle.

Powder X‐ray diffraction (XRD) patterns indicate that the precursor Pt_5_Ce nanoparticles undergo a structural transformation after Co incorporation (Figure 2a). The precursor Pt_5_Ce nanoparticles showed sharp diffraction peaks matching the reference PDF card for Pt_5_Ce (PDF#17‐0071), consistent with a typical CaCu_5_‐type hexagonal phase (Figure S6a) [19]. EDS mapping showed that Ce and Pt distribute uniformly over the particle (Figure S6b). Following Co diffusion, the XRD peaks of Pt_5_(Ce)Co@Pt transformed significantly and aligned closely with simulated L1_2_ Pt_5_Co patterns, confirming a structural rearrangement toward a face‐centered cubic (fcc)‐derived L1_2_ type intermetallic phase. A noticeable shoulder peak near the (111) reflection suggests the presence of multiphase components. Rietveld refinement was conducted on the XRD pattern of Pt_5_(Ce)Co@Pt (Figure S7), indicating that the diffraction features can be reliably described using Pt‐, Pt_3_Co‐, and Pt_5_Ce‐based crystallographic components (R_wp_ = 4.75%, χ^2^ = 1.23). The refinement reveals that the Pt_3_Co‐like phase dominates the sample (68.8 wt.%), accompanied by residual Pt_5_Ce‐like phase (19.2 wt.%) and a minor fraction of metallic Pt (12 wt.%). These results suggest that, after Co incorporation and acid leaching, the Pt_5_Ce precursor undergoes substantial structural transformation into a mixed and heterogeneous Pt‐Co‐Ce system rather than remaining as a single phase.

High‐angle annular dark‐field scanning transmission electron microscopy (HAADF‐STEM) imaging reveals a well‐defined multilayer architecture in the Pt_5_(Ce)Co@Pt nanoparticles (Figure 2b). The particles show distinct contrast variations from center to surface, consistent with a dense core, an ordered intermetallic sublayer, and a Pt‐rich shell. The green dash circles the core region, where it shows atomic columns with varying brightness aligned in parallel rows, indicating partial ordering. The intensity in HAADF‐STEM images is approximately proportional to the square of the atomic number (Z^2^), providing insight into the atomic composition of the sample [40]. The brightest columns, marked by blue spheres, correspond to Pt atoms, which dominate the composition. Several larger, less intense sites at cube centers are attributed to Ce atoms (as marked green sphere), consistent with Ce's larger atomic radius and higher Z‐contrast compared to Co. Darker sites likely represent Co atoms in substitutional positions within the Pt‐Ce matrix (as shown at red spheres). A couple of enlarged HAADF‐STEM images of the core area are displayed in Figure S8. The presence of Co in the vicinity of Ce suggests a solid‐solution or intermetallic‐like configuration, where Ce and Co coexist within a Pt matrix, rather than complete phase segregation. Moving outward, as shown by the red circle in Figure 2b, the intermediate region exhibits an ordered structure resembling the L1_2_‐type phase, with chemically ordered structures where Co occupies corner or face‐centered sites coordinated by Pt atoms. [41, 42, 43] This L1_2_‐type sublayer supports the proposed mechanism of Co diffusion into the Pt_5_Ce precursor, where CaCu_5_‐type hexagonal lattice structure transited into parallel L1_2_‐type structure, and formed a mixed and heterogeneous Pt‐Co‐Ce system. The outermost region, as shown by the blue circle in Figure 2b, exhibits uniform, intense contrast indicating high Pt concentration with minimal Co or Ce incorporation. Clear, continuous lattice fringes here confirm a well‐ordered crystalline Pt shell structure. [34, 41, 44]

Energy‐dispersive X‐ray spectroscopy (EDS) supports the multilayer configuration (Figure 2c). Pt distributes throughout the particle, while Co concentrates in a ring‐like subsurface region, confirming successful Pt‐Co sublayer formation. Ce remains near the particle center, indicating partial displacement during thermal treatment. The combined elemental map shows clear spatial separation: Ce‐rich core, Co‐enriched sublayer, and Pt‐rich shell. To further quantify this spatial distribution, a STEM‐EDS line scan was performed across a representative nanoparticle (Figure 2d) along the arrow direction in Figure 2e. The line profile shows that the Pt signal remains relatively uniform across the entire particle, while the Co signal exhibits a pronounced maximum in the intermediate region, corresponding to a subsurface layer. In contrast, the Ce signal is primarily localized toward the particle center with significantly lower intensity at the outer regions. This spatially resolved compositional gradient is consistent with the EDS mapping results and further confirms the formation of a multilayer architecture. In addition, EDS spectra from selected regions provide quantitative confirmation (Figure 2e). The core region preserved most of the Ce, consistent with a Pt_3_(CeCo)‐type environment. The intermediate region shows 81 at.% Pt, 15 at.% Co, and 4 at.% Ce, corresponding to a Pt_5_Co‐like sublayer, while the near‐surface region is nearly pure Pt (as shown by inserted bars in Figure 2e). A schematic illustration of the Pt_5_(Ce)Co@Pt nanoparticle is presented in Figure 2f, summarizing the multilayer structure derived from structural and compositional analyses. The nanoparticle consists of a Ce‐rich core, surrounded by a Co‐enriched Pt_5_Co‐like sublayer, and an outer Pt‐rich shell. This hierarchical architecture reflects the site‐dependent incorporation of Co into the Pt_5_Ce precursor and the thermodynamically driven redistribution during annealing, which supports the design principle of coupling a strain‐inducing sublayer with a catalytically active Pt shell, further giving rise to the strain‐engineered configuration responsible for the enhanced catalytic performance.

### 2.3 Strain Effect Analysis and ORR Electrochemical Performance

To investigate the strain fine‐tuning effects, Pt_5_(Ce)Co@Pt is compared with Pt_3_Co@Pt, and PtCo@Pt with the same level particle size distribution. HAADF‐STEM images of Pt_5_(Ce)Co@Pt, Pt_3_Co@Pt, and PtCo@Pt nanoparticles show distinct lattice planes, enabling direct measurement of interplanar spacings along the (111), (110), and (100) crystallographic directions (Figure 2g–i). Inverse Fast Fourier Transform (IFFT) analysis for each alloy appears in Figures S9–S11, with corresponding *d*‐spacing values summarized in Table S2. The measured *d*‐spacings reveal systematic lattice contraction with increasing Co content. Pt_5_(Ce)Co@Pt shows the largest spacings: 0.232 nm (111), 0.277 nm (110), and 0.391 nm (100). These values decrease progressively through Pt_3_Co@Pt (0.224, 0.272, and 0.386 nm) to PtCo@Pt (0.212, 0.232, and 0.354 nm). This systematic lattice contraction with higher Co content agrees excellently with the DFT‐predicted surface strain values. Increasing Co content creates stronger compressive strain, contracting the surface lattice (Table 1). These TEM measurements directly validate the theoretical strain predictions. Pt_5_(Ce)Co@Pt shows minimal compression (−1.24%), while Pt_3_Co and PtCo exhibit increasingly severe compression (−2.22% and −5.21%, respectively). Importantly, the Pt_5_(Ce)Co@Pt strain level is optimal for shifting *OH binding energy by ∼0.11 eV, placing it near the ORR activity volcano peak (Figure 1c).

**FIGURE 3:**
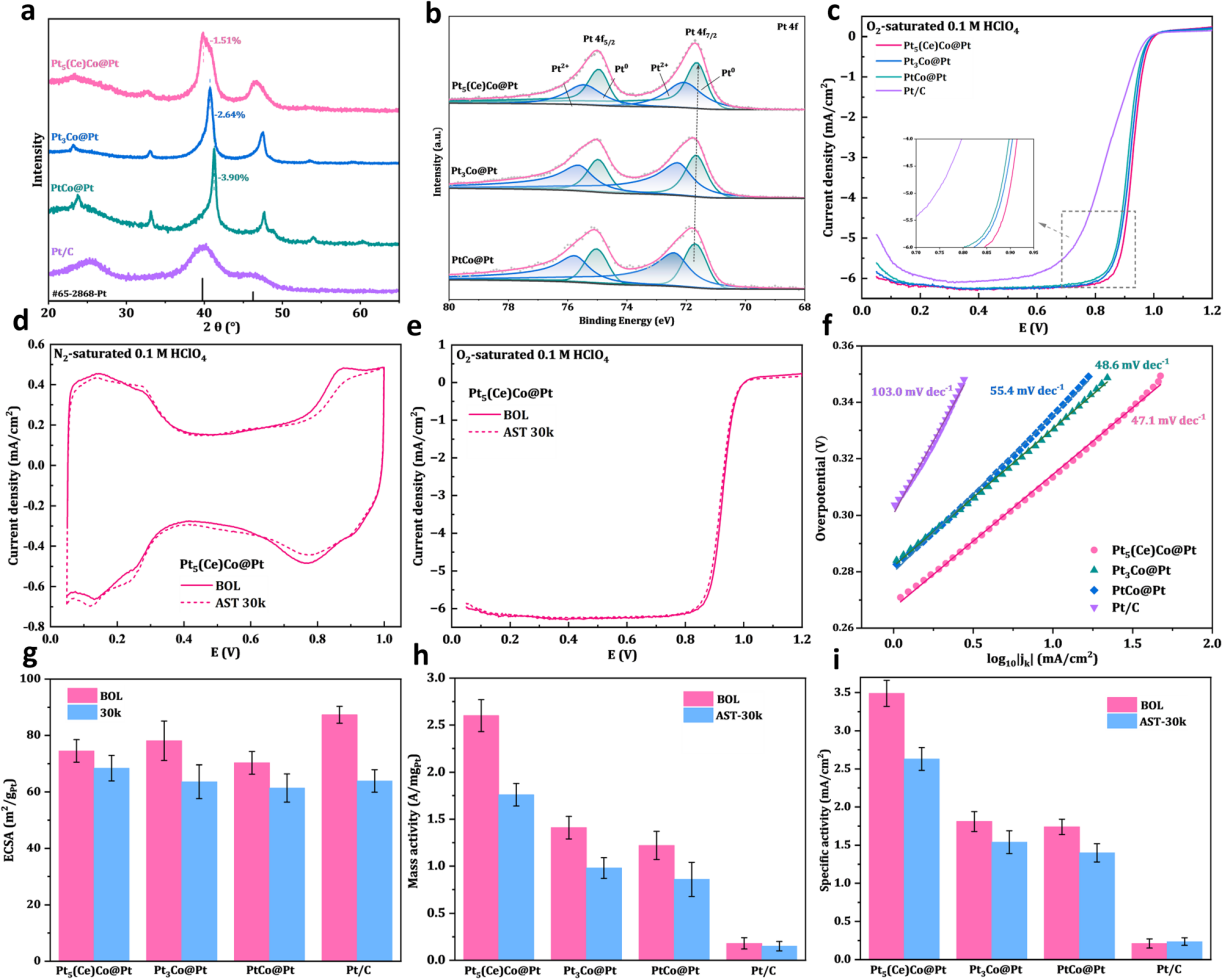
(a) XRD patterns from Pt5(Ce)Co@Pt, Pt3Co@Pt, PtCo@Pt, and Pt/C; (b) XPS patterns of the Pt *4f* peak from Pt_5_(Ce)Co@Pt, Pt_3_Co@Pt, and PtCo@Pt; (c) ORR polarization curves from Pt_5_(Ce)Co@Pt, Pt_3_Co@Pt, PtCo@Pt, and Pt/C. (d) CV curves and (e) ORR polarization curves from Pt_5_(Ce)Co@Pt at BOL and after 30 000 AST cycles; (f) Tafel plot of ORR in RDE testing; (g) ECSA, (h) Mass activity, and (i) specific activity and their loss after 30 000 AST cycles in RDE testing.

Figure 3a shows XRD patterns’ comparison of Pt_5_(Ce)Co@Pt, Pt_3_Co@Pt, PtCo@Pt, and commercial Pt/C (20 wt.% Pt on carbon black). Compared to commercial Pt/C, all alloy samples exhibit a noticeable shift of the (111) peak toward higher 2θ values, indicating the presence of compressive strain due to Co incorporation into the Pt lattice [45, 46]. Among them, Pt_5_(Ce)Co@Pt shows the smallest peak shift, corresponding to a Pt(111) lattice contraction of −1.51%. In contrast, Pt_3_Co@Pt and PtCo@Pt display more substantial shifts, with calculated compressive strains of −2.64% and −3.90%, respectively. These results are in good agreement with the DFT‐predicted strain trend, which identifies Pt_5_Co as inducing an optimal ∼−1.24% surface strain, sufficient to tune *OH binding energy without over‐compressing the lattice. XRD fitting yields consistent lattice constants: 3.854 Å for Pt_5_(Ce)Co@Pt (−1.78%), 3.847 Å for Pt_3_Co@Pt (−1.96%), and 3.800 Å for PtCo@Pt (−3.16%), all relative to bulk Pt/C (3.924 Å). This trend quantitatively confirms the theoretical expectation that increasing Co content introduces progressively stronger lattice contraction [13, 46].

X‐ray photoelectron spectroscopy (XPS) was carried out to gain further insights into the surface electronic structure and oxidation states of the catalysts. Figure 3b displays the Pt *4f* spectra of Pt_5_(Ce)Co@Pt, Pt_3_Co@Pt, and PtCo@Pt, each of which can be deconvoluted into metallic Pt^0^ and oxidized Pt^2+^ components [14, 47, 48]. Notably, a progressive positive shift in the Pt *4f_7/2_* peak position of metallic Pt^0^ was observed with increasing Co content: 71.55 eV for Pt_5_(Ce)Co@Pt, 71.68 eV for Pt_3_Co@Pt, and 71.91 eV for PtCo@Pt. This monotonic increase in binding energy reflects a systematic downshift of the Pt *d*‐band center due to increasing compressive strain [49, 50, 51]. As the Co content increases, the lattice contracts further, inducing more compressive strain, resulting in a more electron‐deficient Pt surface [52, 53]. This shift in binding energy is consistent with both the DFT‐predicted strain trend and XRD‐derived lattice constants, providing experimental evidence for strain‐induced modification of the electronic structure. In addition to binding energy shifts, the Pt^0^/Pt^2+^ peak area ratio also provides insight into surface composition and catalyst structure. Pt_5_(Ce)Co@Pt exhibits the highest Pt^0^/Pt^2+^ ratio of 1.54, compared to 1.30 for Pt_3_Co@Pt and 1.27 for PtCo@Pt. The enhanced surface metallicity implies fewer surface oxides and a more conductive and catalytically active surface, which can facilitate more efficient *OH adsorption and desorption [50, 51].

Overall, the observed Pt^0^ *4f_7/2_* binding energy shifts across the series match well with the degree of compressive strain quantified from both DFT (−1.24% to −5.21%) (Figure 1b) and XRD measurements (Figure 3a). The small negative shift in Pt_5_(Ce)Co@Pt (∼71.55 eV) suggests a mild compressive strain (compared to Pt_3_Co@Pt and PtCo@Pt), inducing a moderate downshift of the Pt *d*‐band and thus positioning the *OH binding energy within the optimal window (ΔE_*OH_ ≈ 0.11 eV) [14, 15, 47]. In contrast, the larger shifts in Pt_3_Co@Pt and PtCo@Pt (71.68 and 71.91 eV, respectively) correspond to more pronounced strain and *d*‐band perturbation, leading to *OH binding energies that deviate from the optimal range [45, 52, 54]. Since the oxidation states and chemical environments remain comparable across all three alloys, the observed binding energy shifts can be predominantly attributed to lattice strain rather than ligand or charge transfer effects, further validating the DFT conclusion that strain is the dominant factor in tuning *OH adsorption energy and ORR activity.

Electrochemical tests were performed to assess the ORR catalytic performance, as well as to further validate these structural insights. As shown in Figure 3c, Pt_5_(Ce)Co@Pt displayed the highest ORR performance in rotating disk electrode (RDE) testing under O_2_‐saturated 0.1 M HClO_4_. At 0.9 V (vs. RHE), it delivered a mass activity (MA) of 2.6 A·mg_Pt_^−1^, nearly doubling that of Pt_3_Co@Pt (1.41 A·mg_Pt_^−1^) and PtCo@Pt (1.22 A·mg_Pt_^−1^), and significantly higher than that of commercial Pt/C (0.18 A·mg_Pt_^−1^), as shown in Figure 3h. Their calculated specific activity (SA) follows the same trend, as summarized in Figure 3i. This enhanced activity is also reflected in the half‐wave potential (*E*_1/2_). Specifically, Pt_5_(Ce)Co@Pt exhibited an *E*_1/2_ of 0.93 V, compared to 0.92 V for Pt_3_Co@Pt and 0.91 V for PtCo@Pt. This positive shift in *E*_1/2_ reflects improved reaction kinetics and correlates with the more optimal *OH binding energy induced by the moderate compressive strain in Pt_5_(Ce)Co@Pt, aligning well with the DFT‐predicted strain‐activity relationship and confirming that Pt_5_(Ce)Co@Pt is positioned closest to the peak of the ORR activity volcano. The RDE‐measured activity trend, Pt_5_(Ce)Co@Pt > Pt_3_Co@Pt > PtCo@Pt > Pt/C, therefore, provides strong experimental validation of the DFT‐predicted strain‐activity relationship.

The stability of the Pt_5_(Ce)Co@Pt catalyst was evaluated through accelerated stress testing (AST) over 30 000 potential cycles. Figure 3d,e, and Figure S12 show the cyclic voltammetry (CV) and ORR polarization curves before and after AST cycles of Pt_5_(Ce)Co@Pt, Pt_3_Co@Pt, PtCo@Pt, and commercial Pt/C. While only a modest decline in activity was observed, Pt_5_(Ce)Co@Pt still retained 69% of its initial MA (2.6 to 1.8 A∙mg_Pt_^−1^), remaining higher than the initial activities of Pt_3_Co@Pt and PtCo@Pt. Figure 3g–i quantify the activity degradation, showing that Pt_5_(Ce)Co@Pt undergoes a 31% loss in MA, 33% loss in SA, and only 8% loss in ECSA after 30 000 AST cycles. This level of degradation is on par with that of Pt_3_Co@Pt (30% loss in MA) and PtCo@Pt (29% loss in MA), despite the much higher activity of Pt_5_(Ce)Co@Pt. It indicates that a Ce‐rich core may provide stabilization to the Pt_5_Co‐like sublayer. Specifically, the Ce template not only enables the formation of this otherwise inaccessible structure of Pt_5_Co but also suppresses atomic diffusion and structural collapse, thereby preserving the catalyst's robustness under extended operation. A comparison with other Pt‐Co‐related alloy catalysts reported in the most recent five years was summarized in Table S3. Among all the Pt‐Co related catalysts, Pt_5_(Ce)Co@Pt stands out in both activity and stability performance, which proves that the strain‐guided design and the application of the Ce template enable high activity without sacrificing stability, a critical advantage in practical applications.

Kinetic analysis via Tafel plots (Figure 3f) further validates the theoretical predictions. Pt_5_(Ce)Co@Pt exhibited the lowest Tafel slope (47.1 mV·dec^−1^), followed by Pt_3_Co@Pt (48.6 mV·dec^−1^), PtCo@Pt (55.4 mV·dec^−1^) and Pt/C (103.0 mV·dec^−1^). This trend reflects faster reaction kinetics and is fully consistent with the DFT‐calculated *OH adsorption strengths. As surface binding approaches the optimal range (ΔE_*OH_ ≈ 0.10–0.12 eV), the energy barrier for key ORR steps is minimized, resulting in steeper current‐voltage response and reduced Tafel slope. The linear behavior across the low‐overpotential region also confirms that all catalysts operate under kinetic control. Thus, both activity and kinetic trends reinforce the mechanistic framework derived from DFT.

### 2.4 Membrane Assembly Electrode (MEA) Testing

**FIGURE 4:**
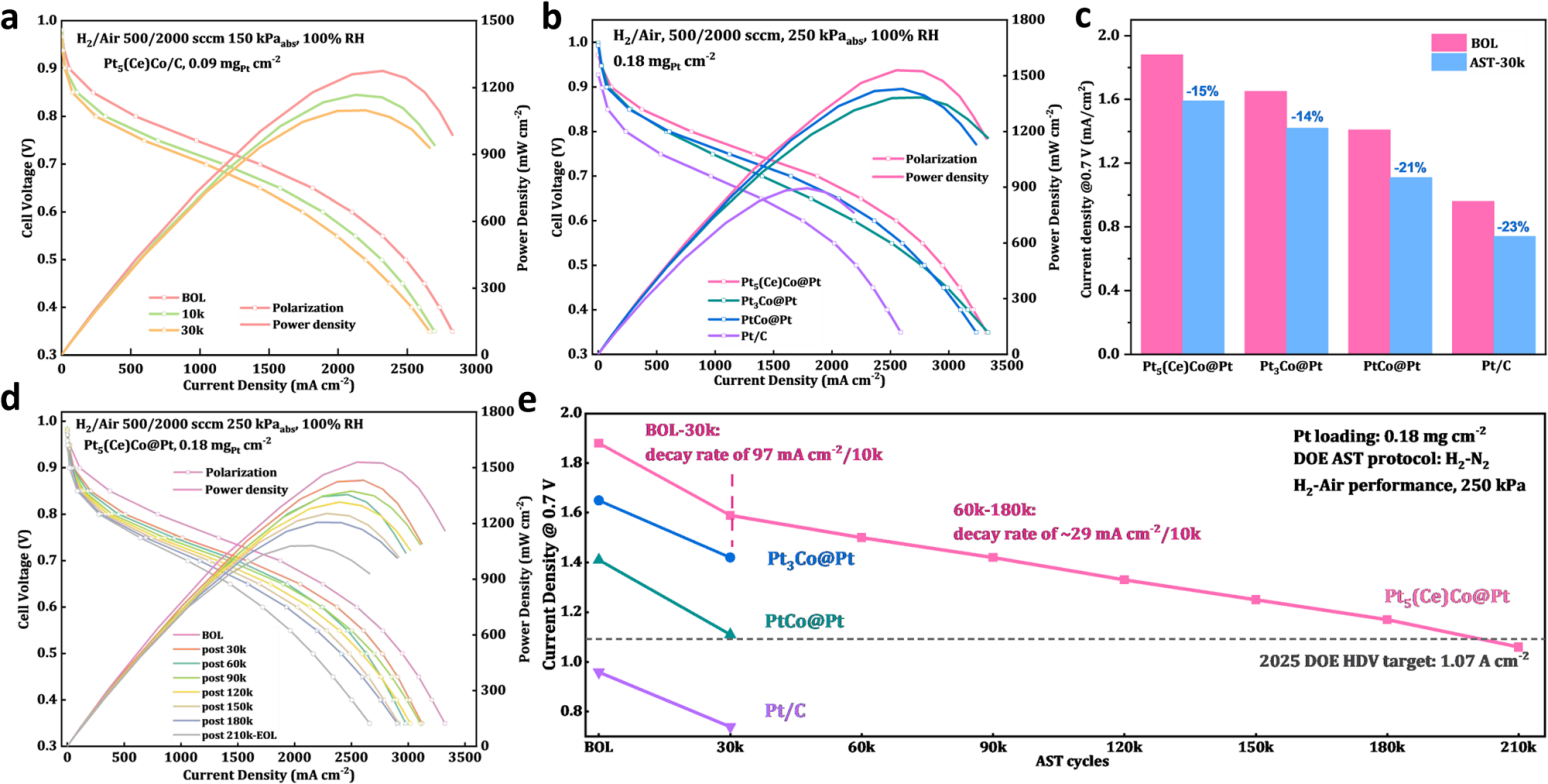
a) H_2_/air polarization curves and power density curves of Pt_5_(Ce)Co@Pt at BOL and after 10,000 and 30,000 AST cycles; Test conditions (LDV): Anode: 30 wt.% JP30S, 0.1 mg_Pt_/cm^2^; Cathode: Pt_5_(Ce)Co@Pt, 0.09 mg_Pt_/cm^2^; Differential Cell, 500/2000 sccm, H_2_/Air, 150 kPa_abs_, operation temperature 80°C, and 100% relative humidity. b) H_2_/air polarization curves and power density curves of Pt_5_(Ce)Co@Pt, Pt_3_Co@Pt, PtCo@Pt, and Pt/C tested at the beginning of life (BOL); c) Current density loss at 0.7 V after 30,000 AST cycles; d) H_2_/air polarization curves and power density curves of Pt_5_(Ce)Co@Pt at BOL and after 30,000 to 180,000 AST cycles. e) Current density retention at 0.7 V during HDV AST testing. Test conditions (HDV): Anode: 30 wt.% JP30S 0.1 mg_Pt_/cm^2^; Cathode: catalysts; 0.18 mg_Pt_/cm^2^; Differential Cell, 500/2000 sccm, H_2_/Air, 250 kPa_abs_, Operation temperature 80°C, and 100% relative humidity.

All the catalysts were also tested in the membrane assembly electrode (MEA) configuration under both heavy‐duty‐vehicle conditions (HDV) and light‐duty‐vehicle conditions (LDV) according to U.S. DOE MEA testing protocol [55, 56]. Under LDV conditions (0.09 mg_Pt_∙cm^−2^, 150 kPa_abs_), Pt_5_(Ce)Co@Pt reaches 0.59 A·cm^−2^ at 0.8 V (Figure 4a) and a mass activity of 1.58 A·mg_Pt_^−1^ at 0.9 V_iR‐corrected_, far exceeding DOE 2025 activity targets (0.3 A·cm^−2^ at 0.8 V and mass activity of 0.44 A·mg_Pt_^−1^ at 0.9 V_iR‐corrected_). Figure 4b presents the H_2_/air polarization and power density curves for the Pt_5_(Ce)Co@Pt, Pt_3_Co@Pt, PtCo@Pt, and Pt/C catalysts in MEA testing under HDV conditions (0.18 mg_Pt_∙cm^−2^, 250 kPa_abs_). Among all the catalysts, Pt_5_(Ce)Co@Pt exhibits the highest performance, achieving a current density of 1.88 A·cm^−2^ at 0.7 V and a mass activity of 0.78 A·mg_Pt_^−1^ at 0.9 V*_iR‐corrected_*. These values are substantially higher than those of Pt_3_Co@Pt (1.65 A·cm^−2^, 0.68 A·mg_Pt_^−2^), PtCo@Pt (1.41 A·cm^−2^, 0.53 A·mg_Pt_^−2^), and commercial Pt/C (20 wt.% Pt on carbon black, 0.96 A·cm^−2^, 0.12 A·mg_Pt_^−2^), confirming that the high intrinsic activity of Pt_5_(Ce)Co@Pt observed in RDE testing translates directly into full‐cell operation. Stability under fuel cell conditions was evaluated by subjecting the MEAs to AST potential cycles following U.S. DOE protocol [57]. The performance from beginning‐of‐life (BOL) to the first 30 000 AST cycles under HDV conditions was compared among all the catalysts. As shown in Figure S13, although all catalysts experience some degree of performance degradation, Pt_5_(Ce)Co@Pt retains a high level of activity after 30 000 AST cycles. The current density at 0.7 V decreased from 1.88 A·cm^−2^ at BOL to 1.59 A·cm^−2^ after 30 000 AST cycles, corresponding to a 15% loss. This level of degradation is comparable to Pt_3_Co@Pt (14% loss) and PtCo@Pt (21%), as summarized in Figure 4c. Likewise, in Figure S14, the mass activity loss for Pt_5_(Ce)Co@Pt (∼44%) remains within the range observed for the other Pt‐Co‐based alloy catalysts, indicating that the multilayer architecture Pt_5_(Ce)Co@Pt nanoparticles achieves enhanced performance without sacrificing stability (Table S4). Long‐term durability was also demonstrated under extended HDV AST cycling till 2 10 000 cycles (Figure 4d): even after 1 80 000 AST cycles, Pt_5_(Ce)Co@Pt retains a current density of 1.17 A·cm^−2^ at 0.7 V, well above the DOE 2025 targets of 1.07 A·cm^−2^ after 90 000 AST cycles at 0.7 V, as shown in Figure 4e. The degradation profile of Pt_5_(Ce)Co@Pt during extended HDV AST cycling follows a two‐stage pattern similar to that observed in our previous MEA durability study [58]. A relatively rapid activity loss occurred during the initial 30 000 AST cycles, which may be attributed to a brief phase of accelerated Ostwald ripening and surface restructuring of smaller particles. Beyond 60 000 AST cycles, however, the degradation rate slows significantly and becomes nearly linear up to 1 80 000 cycles, indicating that the system enters a steady state with greatly suppressed degradation. The decay‐rate analysis (Table S5) reveals that Pt_5_(Ce)Co@Pt loses 15.4% of its activity during the first 30,000 AST cycles, with a current density decay rate of 97 mA∙cm^−2^ per 10 000 AST cycles (5.1%). Its degradation then stabilizes to 29 mA∙cm^−2^ (∼2%) per 10 000 AST cycles in later stages.

**FIGURE 5:**
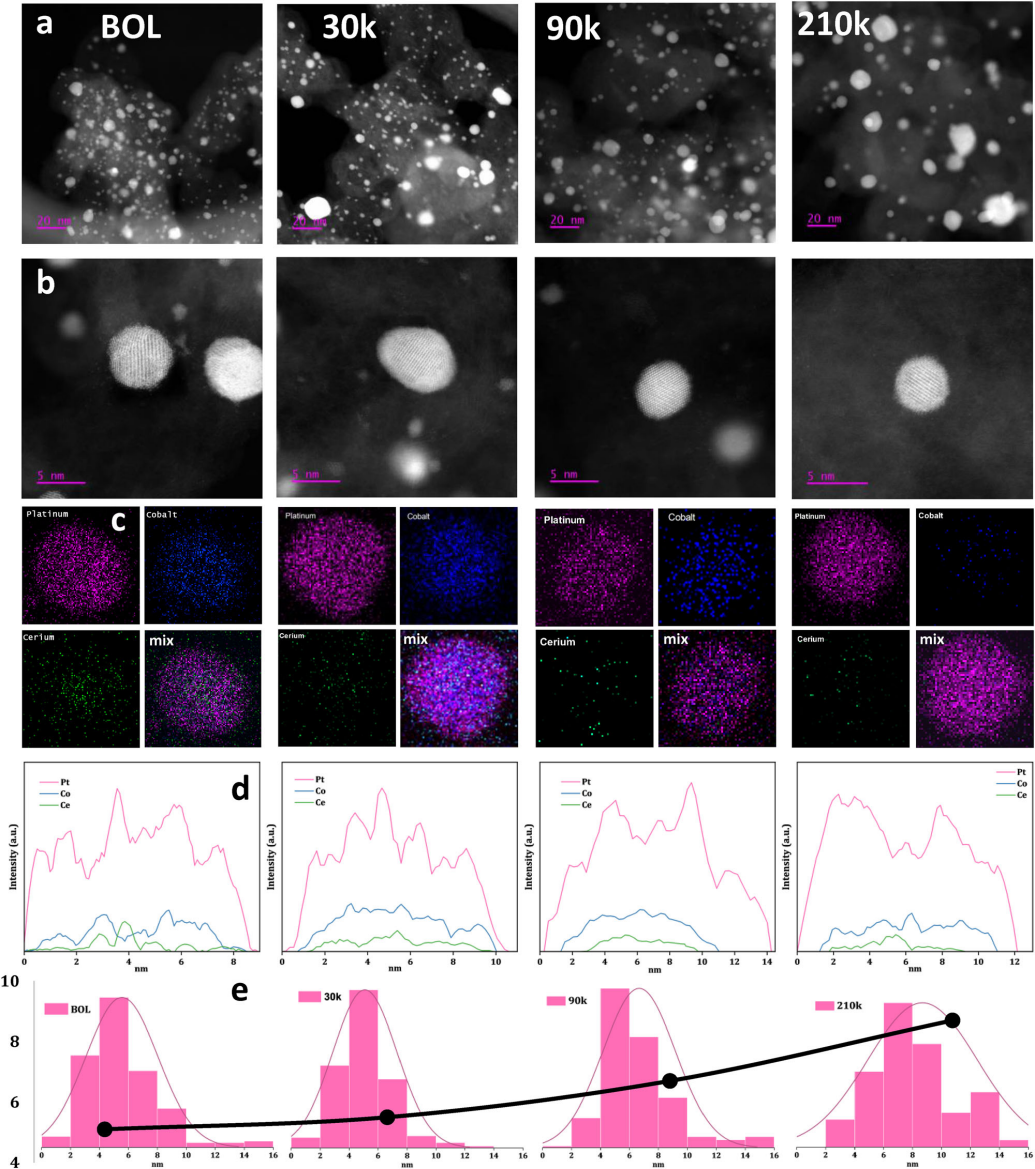
(a) Low‐mag STEM images, (b) HAADF‐STEM images, (c) EDS mapping images, and (d) line scan profiles (e) particle size distributions (black line represents the average particle size trend, 1200 particles in total were counted) of Pt_5_(Ce)Co@Pt particles from beginning of life to after 30 000, 90 000, and 2 10 000 AST cycles under heavy‐duty‐vehicle MEA testing conditions. AST protocol: Anode: 30 wt.% JP30S, 0.1 mg_Pt_/cm^2^; Cathode: Pt_5_(Ce)Co@Pt; 0.18 mg_Pt_/cm^2^; Differential Cell, 50/75 sccm, H_2_/N_2_, 50 kPa_abs_, Operation temperature 80°C, and 100% relative humidity. please *note*: Each row is arranged from left to right as BOL, 30 000, 90 000, and 2 10 000 AST cycles.

To further probe long‐term structural evolution beyond the electrochemical results shown to 1 80 000 AST cycles in Figure 4, an additional 30 000 AST cycles were performed for post‐structural characterization, bringing the total to 2 10 000 AST cycles for the samples analyzed in Figure 5. Several MEA tests were also conducted up to 30 000 and 90 000 AST cycles, respectively, and the corresponding catalysts after cycles were analyzed using HAADF‐STEM and EDS mapping (Figure 5). Low‐magnification STEM images (Figure 5a) from BOL to post 2 10 000 AST cycles reveal the evolution of particle size distribution during AST cycling. As shown in Figure 5e, from BOL to 30 000 cycles, the particle size distribution remains largely unchanged, indicating minimal structural degradation at the early stage. The average particle size has a small increase from 5.1 to 5.5 nm, which implies the accelerated Ostwald ripening and surface restructuring of smaller particles during the first 30 000 AST cycles [59, 60]. However, between 30 000 and 90 000 AST cycles, a noticeable reduction in the population of smaller nanoparticles (<4 nm) is observed with average size increasing to 6.7 nm (Figure 5e), suggesting combined particle migration and Ostwald ripening of smaller particles during long‐term operation [58]. With further cycling from 90 000 to 2 10 000 AST cycles, the particle size distribution shifts toward larger sizes with an average of 8.7 nm, accompanied by a significant decrease in the number of particles within the 2–6 nm range (Figure 5e). This trend indicates continued particle coarsening, consistent with classical degradation mechanisms in Pt‐based catalysts [58, 59, 61]. Despite these changes, the overall structural integrity of the catalyst remains well preserved. HAADF‐STEM images and STEM‐EDS mapping (Figure 5b,c) show that the multilayer architecture is retained even after prolonged cycling. After 30,000 AST cycles, the characteristic Ce‐containing core and Co‐enriched sublayer beneath a Pt‐rich shell are still observable. With increasing cycling, the Pt‐rich shell becomes thicker, suggesting surface reconstruction and Pt redeposition during operation. Importantly, even after 2 10 000 AST cycles, there are particles maintaining the core–shell structure, although with reduced compositional contrast. This persistence of the multilayer structure suggests that the Ce‐templated framework provides structural stability, mitigating complete phase collapse during long‐term operation. From the line scan in Figure 5d, the gradual loss of Co and Ce from the outer regions, combined with Pt surface enrichment, leads to a stabilized configuration that suppresses further rapid degradation.

These observations support a two‐stage degradation mechanism observed in Figure 4e. In the initial stage (30 000 AST cycles), the catalyst undergoes limited structural change. In the intermediate stage (30 000–90 000 AST cycles), smaller particles dissolve or merge, leading to a redistribution of particle sizes. In the later stage (>90 000 AST cycles), the system reaches a quasi‐stable state characterized by a thicker Pt shell and a preserved core–shell structure, resulting in significantly reduced degradation rates. The improved long‐term retention could be associated with the Ce‐templated architecture, where the Ce‐rich core may enhance the structural and thermodynamic stability of the surrounding Pt_5_Co‐like sublayer. Although further investigation is needed to confirm the precise mechanism, the presence of Ce likely contributes to reduced atomic mobility and sustained integrity of the active structure under prolonged operation.

These MEA results reinforce the trends established by half‐cell RDE testing. In both platforms, Pt_5_(Ce)Co@Pt delivers the highest ORR activity, confirming that the DFT‐guided design, specifically the introduction of an optimal ∼−1.24% compressive strain via a Pt_5_Co‐like sublayer, successfully tunes *OH adsorption energy to the ideal range (ΔE_*OH_ ≈ 0.11 eV). Electrochemical measurements reveal a clear volcano‐type trend in ORR activity with the DFT‐calculated ΔE_*OH_ across the series of catalysts as anticipated (Figure 1). The consistent performance advantage in both RDE and MEA setups supports the mechanistic rationale that strain engineering at the atomic level, when combined with thermodynamically stable Ce‐containing cores and Pt‐rich shells, enables Pt_5_(Ce)Co@Pt to simultaneously achieve record‐high ORR activity and outstanding durability, underscoring rare‐earth templating as a powerful route to design high‐performance PEMFC catalysts.

## 3 Conclusion

Through a combination of first‐principles modeling and experiments, we have established a rational pathway to design Pt‐based catalysts for ORR by correlating lattice strain and catalytic activity. DFT calculations showed that a Pt_5_Co‐like core induces an optimal compressive strain of −1.24% on a Pt(111) surface, yielding an *OH binding energy shift on the order of 0.107 eV and placing the catalyst at the peak of the ORR activity volcano. Given that Pt_5_Co is not a thermodynamically stable bulk phase, we realized this structure by employing Ce to form a structural template. The synthesized Pt_5_(Ce)Co@Pt nanoparticles exhibit a multilayered architecture with a Ce‐rich stabilizing core, a Pt_5_Co‐like sublayer, and a Pt‐rich shell. The strain effect trend was compared experimentally and is consistent with DFT simulations. Electrochemical testing validated the theoretical prediction. Pt_5_(Ce)Co@Pt demonstrated record‐high ORR activity, superior to Pt_3_Co@Pt, PtCo@Pt, and commercial Pt/C. Notably, the catalyst exhibited exceptional durability: maintaining 1.17 A·cm^−2^ at 0.7 V after 1 80 000 MEA AST cycles under MEA HDV protocol, well beyond the DOE HDV 2025 target of 1.07 A·cm^−2^ after 90 000 cycles. This durability highlights the thermodynamical and structural stabilizing role of the Ce‐rich core, which may suppress atomic diffusion and preserve the Pt_5_Co‐like active sublayer under extended operation.

Overall, this work demonstrates a viable strategy for catalyst optimization by fine‐tuning lattice strain, which unites DFT‐guided strain engineering with rare‐earth templating to achieve catalysts that are both highly active and highly durable. Ultimately, tuning the alloy composition at the atomic level as predicted by theory enabled us to optimize the surface chemistry for ORR. Looking forward, exploration of size‐strain‐composition tradeoffs may further expand the design space for high‐performance, cost‐effective electrocatalysis.

## Author Contributions

Q.Z., H.Z., P.L., and J.X. proposed the concept and designed all experimental and theoretical studies. Q.Z., G.Z., and Y.L. carried out materials synthesis and characterization, electrochemical measurements, and fuel cell tests. S.J., E.O.O., and E.A.S. conducted electron microscopy studies. H.Z. and P.L. performed the DFT calculation. B.E.V.P. and G.M.F. conducted ICP‐AES studies. H.G., B.Z., Y.L., and X.Y. conducted XPS testing. C.L. made a figure revision. Q.Z., J.X., H.Z., P.L., S.J., and E.A.S. wrote the manuscript.

## Conflicts of Interest

The authors declare no conflict of interest.

## Supporting information




**Supporting File**: adma73269‐sup‐0001‐SuppMat.docx.

## Data Availability

The data that support the findings of this study are available from the corresponding author upon reasonable request.
