# Supplementary material for: Theory Guided Fine‐Tune of Strain Effects in Pt Ternary Alloy via Rare Earth Templating: Achieving High Performance PEMFCs Catalysts
Source: Adv Mater. 2026 May 18;38(35):e73269. doi: 10.1002/adma.73269 (PMC13288199; doi:10.1002/adma.73269)
Supplement: Supplementary file 1 — Supporting File: adma73269‐sup‐0001‐SuppMat.docx. [file ADMA-38-e73269-s001.pdf]

Theory Guided Fine-Tune of Strain Effects in Pt Ternary Alloy via Rare Earth Templating: Achieving High Performance PEMFCs Catalysts

Qi Zhang, Hong Zhang, Sungho Jeon, Erika Ortega Ortiz, Brooke E. Vander Pas, Guangqi Zhu, Yi-Kai Lien, Chenzhao Li, Huayu Guo, Baixu Zhu, Yaroslav Losovyj, Gabriel M. Filippelli, Xingchen Ye, Eric A Stach, Ping Liu*, and Jian Xie**

Q. Zhang, H. Zhang, S. Jeon
These authors contributed equally to this work.

Q. Zhang, G. Zhu, C. Li, J. Xie*
School of Mechanical Engineering, Purdue University, West Lafayette, IN 47907, USA
E-mail: jxie@purdue.edu

H. Zhang, P. Liu*
Department of Chemistry, Stony Brook University, Stony Brook, NY 11794, USA
E-mail: pingliu3@bnl.gov

S. Jeon, E. O. Ortiz, E. A. Stach*
Department of Materials Science and Engineering, University of Pennsylvania, Philadelphia, PA 19104, USA
E-mail: stach@seas.upenn.edu

B. E. Vander Pas, G. M. Filippelli
Department of Earth Science, Indiana University, Indianapolis, IN 46202, USA

Y. Lien, J. Xie*
School of Materials Engineering, Purdue University, West Lafayette, IN 47907, USA
E-mail: jxie@purdue.edu

H. Guo, B. Zhu, Y. Losovyj, X. Ye
Department of Chemistry, Indiana University, Bloomington, IN 47405, USA

P. Liu*
Chemistry Division, Brookhaven National Laboratory, Upton, NY 11973, USA
E-mail: pingliu3@bnl.gov

Experiments:

DFT calculations. Spin polarized DFT calculations using Vienna Ab initio Simulation Package (VASP)[1-3] with Perdew-Burke-Ernzerhof (PBE)[4] functionals were performed. The DFT optimized face center cubic (FCC) Pt unit cell with lattice constant of 3.975Å was modified to form FCC like Pt_xCo (x = 1, 3, 5) units, where the Pt_5Co is approximated by a $Pt_{27}Co_5$ supercell (**Figure 1a**). The formation energy of Pt_xCo units $E^u = E_{PtxCo} - x * E_{Pt} - E_{Co}$ where E_{Pt} and E_{Co} is the energy per atom in Pt face centered cubic (FCC) unit and Co hexagonal close pack (HCP) unit (**Figure S4**).

Each unit cell is used to create a 2×2 slab model contains 3 layer of Pt(111) shell supported by 3 layer of base material to represent the core@shell structure (**Figure S1 and S2**). The slab is extended to infinite surface in periodic condition and 15 Å vacuum is placed above the surface to ensure a minimum of 12 Å of space when intermediate molecules are adsorbed, and dipole correction along the direction of vacuum is included. The bottom three layers of bulk material is frozen to represent the core structure underneath, the top three layers along with adsorbed *OH are allowed for relaxation. Atomic optimizations were initialized using the Conjugate Gradient ionic relaxation algorithm[5] then converged with the RMM-DIIS ionic relaxation algorithm[6] with a plane wave cutoff energy of 400 eV. A 7×7×1 Monkhorst-Pack grid and first order Methfessel-Paxton with smearing width of 0.2 eV were used to integrate over Brillouin zone. An electronic convergence level of $1x10^{-6}$ eV was employed to obtain convergence of the electronic structure, and ionic relaxation was activated and satisfied until the Hellman-Feynman force was less than 0.02 eV/Å on each ion. The binding energy of *OH was calculated $E_{*OH} = E_{*OH/slab} - E_{clean\ slab} - E_{OH}$ (**Figure 1a**).

To calculate the substitution energy of Ce by Co, the process can be represented as:

$$Pt_5Ce_{(s)} + Co^{2+}{}_{(aq)} + 2H_2O_{(l)} \rightarrow Pt_5Co_{(s)} + CeO_{2(s)} + 4H^+{}_{(aq)} + 2e^-$$

The chemical potentials of $Pt_5Ce_{(s)}$, $2H_2O_{(l)}$, $Pt_5Co_{(s)}$, and $CeO_{2(s)}$ are computed as $\mu = E_0 + ZPE + \Delta H_T - TS$, where the total energy (E_0) is the DFT calculated energy. Zero Point Energy (ZPE) and the entropy S at temperature T were determined according to the NIST Computational Chemistry Comparison and Benchmark Database (CCCBDB) [7] at the same level of theory used in our calculations. The enthalpy (ΔH_T) terms was obtained from NIST-JANAF thermochemical tables [8]. As no external potential applied during the synthesize (U = 0 vs RHE), the chemical potential of $4H^+{}_{(aq)} + 2e^-$ is simplified using Computational Hydrogen Electrode (CHE) bookkeeping as equivalent as $2H_{2(g)}$. Therefore, the free energy change of Ce to Co substitution is:

$$\Delta G = (\mu(Pt_5Co_{(s)}) + \mu(CeO_{2(s)}) + 2\times\mu(H_{2(g)})) - (\mu(Pt_5Ce_{(s)}) + \mu(Co^{2+}{}_{(aq)}) + 2\times\mu(H_2O_{(l)}))$$

The energetic reference for aqueous cobalt ions was estimated using the equilibrium of the redox couple:

$$Co^{2+}{}_{(aq)} + 2e^- \rightleftharpoons Co_{(s)}$$

The standard reduction potential is $E^0(Co^{2+}/Co) = -0.277V$ vs SHE [9-11]. At the ternary catalyst synthesize condition of U = 0 V vs RHE and pH = 7, the electron chemical potential corresponds to $U_{SHE} = U_{RHE} - 0.059pH = -0.414$ V. The equilibrium potential of the Co redox couple $[Co^{2+}]$ = 1.8 mM is obtained from the Nernst equation:

$$E_{eq} = E^0(Co^{2+}/Co) + \frac{K_B T}{2F}\ln[Co^{2+}]$$

where K_B is the Boltzmann constant = 8.617333262 × 10^{-5} eV, and F is the Faraday constant that corresponds to 1 e × 1 V = 1 eV. This gives E_{eq} = -0.358 V vs. SHE at 298.15 K. The chemical potential difference between Co^{2+}(aq) and metallic Co energy E_0(Co) from DFT calculation is:

$$\mu(Co^{2+}_{(aq)}) = \mu(Co_{(s)}) - nF(U_{SHE} - E_{eq}) = \mu(Co_{(s)}) + 0.112 \text{ eV at n=2}$$

To calculate the free energy change of substitution of slab models, the μ(Pt_5Co@Pt) is the total energy of a 3-layer 4×4 slab of Pt(111) shell supported on Pt_5Co lattice (**Figure S2A**), μ(Pt_5Ce@Pt) is the total energy of a 3-layer 4×4 slab of Pt(111) shell supported on Pt_5Ce lattice and a *Kagomé* interlayer[12] (**Figure S2B**).

Kinetic Volcano Plot Construction. The ORR volcano plot was constructed following the framework established by Nørskov and co-workers [13], which correlates the catalytic activity with the adsorption free energy of oxygen intermediates, particularly *OH. The descriptor used in this work is the relative *OH adsorption energy.

$$\Delta E_{*OH} - \Delta E_{*OH}^{Pt(111)}$$

where Pt(111) is used as the reference surface.

The adsorption energies of *OH on different Pt_xCo@Pt surfaces were obtained from DFT calculations as described above. Based on the established scaling relationships for ORR intermediates, *OH binding energy serves as an effective descriptor for catalytic activity.

The kinetic current density (j_k) was derived using the microkinetic model proposed by Nørskov et al.,[13] where the rate-determining step is associated with *OH removal. The activity is expressed as:

$$j_k \propto \exp\left(-\frac{\Delta G_{*OH} - \Delta G_{*OH}^{opt}}{k_B T}\right)$$

where ΔG_{*OH} is the adsorption free energy of *OH, ΔG_{*OH}^{opt} corresponds to the optimal binding energy at the top of the volcano, k_B is the Boltzmann constant, T is the temperature.

The calculated kinetic current density was normalized to Pt(111) and plotted in logarithmic scale:

$$\log\left(\frac{j_k}{j_{k,Pt}}\right)$$

to construct the volcano relationship.

The optimal activity region corresponds to a *OH binding energy approximately 0.10–0.12 eV weaker than Pt(111), consistent with previous reports. The volcano curve shown in **Figure 1c** is adapted from literature trends and serves as a guide to illustrate the relationship between *OH binding strength and catalytic activity.

Experimental data points from this work were plotted using measured current density values at 0.9 V vs. SHE, while literature data points were collected from reported Pt-based catalysts with available activity and adsorption energy data.

Synthesis and Characterizations of the alloys

Synthesis of Binary Pt_5Ce/C. 65 mg (0.13 mmol) $H_2PtCl_6·6H_2O$, 72.6 mg (0.195 mmol) $CeCl_3·7H_2O$, and 519 mg CN_2H_2 were mixed in an agate mortar and pestle, and fully ground and mixed, until an orange slurry was obtained. Then, 100 mg ketjen carbon black (KB) (EC-300J) was added into the slurry and thoroughly ground. The obtained dense black powder was transferred into a quartz boat. This step was carried out in a glove box filled with Ar. Next, the quartz boat with black power in it was transferred to a tube furnace, that was sealed tightly and purged with 7% H_2 in Ar forming gas for three times. The heating program is 10 °C/min from room temperature to 180 °C, holding on 180 °C for 30 min; then 10 °C/min from 180 °C to 750 °C, and maintaining at 750 °C for 3 h, finally cooling down naturally.

Synthesis of Ternary Pt_5Ce-Co@Pt. The binary alloy sample prepared above was dispersed in 20 mL DI-water in a 100 mL round-bottom flask and sonicated for 15 min. 5.8 mg (0.045 mmol) anhydrous $CoCl_2$ was dissolved in 5 mL DI-water in a small vial, and slowly transferred into the binary alloy suspension using a pipette and sonicated for 2 hours. Next, the round-bottom flask was fixed on the Rotary Evaporator, with rotating speed 100 rpm, water tank temperature 60 °C. After the evaporation process was complete, the black powder sticking on the inner surface of round-bottom flask was scraped off and transferred to a quartz boat. After transferring quartz boat to a tube furnace, the tube was sealed tightly and purged with 7% H_2 in Ar forming gas at least three times. The heating program is 8 °C/min from room temperature to 400 °C, holding on 400 °C for 2 h; then 8 °C/min from 400 °C to 650 °C, maintaining at 650 °C for 6 h, finally cooling down naturally. The obtained catalyst was acid washed using 0.5 M H_2SO_4, at 70 °C for 3 h. Finally, the sample was washed using DI-water to obtain the final product.

Synthesis of Pt_3Co@Pt and PtCo@Pt. The synthesis of the two Pt-Co alloys was following the same procedure of Pt_5(Ce)Co@Pt based on Pt:Co stoichiometry in final ordered intermetallic structure but without Ce precursor.

Physical Characterization. The detailed Pt loadings for the alloy catalysts were determined using Inductively Coupled Plasma Atomic Emission Spectroscopy (ICP-AES, Teledyne Leeman Labs). The powder X-ray diffraction (XRD) spectrum was performed on a Broker AXS Discover D8 model at 3 kW (Cu Kα), with scan step of 2 s/step. X-Ray Photoelectron Spectroscopy (XPS) testing was performed on PHI Versaprobe II XPS using an Al Ka radiation (hν = 1486.6 eV) beam (100 mm, 25 W).

TEM analysis. HAADF-STEM imaging and EDS mapping were carried out on a JEOL NEOARM S/TEM operated at 200 kV. A 6C probe size and a 40 μm condenser aperture were employed, yielding a probe current of approximately 62.5 pA. The convergence semi-angle was set to 25 mrad. Data acquisition and analysis were conducted using Gatan DigitalMicrograph software.

Electrochemical Testing

Rotating Disk Electrode (RDE) Testing Setup. Electrochemical measurements were performed in a typical three-electrode system, utilizing a glassy carbon (0.196 cm^2) rotating disc electrode (RDE, Pine Research Instrument) as working electrode, Pt wire (Pine Research Instrument) and a reversible hydrogen electrode (RHE, HydroFlex) as counter and reference electrodes, respectively. All the potentials in this work were reported with respect to RHE. Electrochemical measurements

were carried out using an electrochemical potentiostat (VSP, Bio-Logic) at room temperature. Catalyst inks were prepared by ultrasonically dispersing 5 mg catalysts into a mixture solution of 4 mL DI water, 1mL 2-propanol and 13 μL Nafion solution (5 wt.% D520). The ink was uniformly deposited onto the polished glassy carbon electrode with a designed Pt loading of 12 μg·cm^{-2} and then dried in air at room temperature.

RDE Testing Protocols. All electrochemical tests were performed in 0.1 mol·L^{-1} $HClO_4$ electrolytes at room temperature. Prior to electrochemical tests, the electrodes were activated by cycling potential between 0.05 and 1.20 V at 500 mV·s^{-1} for 50 cycles in N_2-saturated electrolytes. Cyclic voltammograms (CVs) were measured by scanning potential from 0.05 to 1.00 V at 50 mV·s^{-1} in N_2-saturated electrolytes. ORR polarization curves were recorded in O_2-saturated electrolytes at a rotating rate of 1600 rpm and a sweep rate of 20 mV·s^{-1}, with iR correction. Catalyst durability tests were performed by conducting 30,000 potential cycles between 0.60 and 0.95 V (trapezoidal wave with 0.5 s rise time and 2.5 s hold time) in N_2-saturated electrolytes.

Electrochemical Surface Area (ECSA) Calculation. The ECSA was determined from the hydrogen underpotential deposition (H_{upd}) region of cyclic voltammetry (CV) curves recorded in N_2-saturated 0.1 M $HClO_4$ at a scan rate of 50 mV·s^{-1}. The integrated charge (Q) was obtained by subtracting the double-layer capacitance and integrating the current over the H_{upd} potential region:

$$ECSA = \frac{Q}{0.21\, mC \cdot cm_{Pt}^{-2} \times m_{Pt}}$$

where Q is in mC, $0.21\, mC \cdot cm_{Pt}^{-2}$ is the charge density required for monolayer hydrogen adsorption on Pt, m_{Pt} is the mass of Pt loaded on the electrode (mg). The ECSA is reported in $m^2 \cdot g_{Pt}^{-1}$.

Mass Activity (MA) Calculation. The kinetic current density (j_k) was derived from the measured current density (j) and the diffusion-limited current density (j_d) using the Koutecky–Levich equation:

$$\frac{1}{j} = \frac{1}{j_k} + \frac{1}{j_d}$$

The MA was then calculated as:

$$MA = \frac{j_k(0.9\, V\, vs. RHE)}{m_{Pt/area}}$$

where j_k is in A·cm^{-2}, $m_{Pt/area}$ is in mg·cm^{-2}. The MA is reported in A·mg_{Pt}^{-1}.

Specific Activity (SA) Calculation. Specific activity was calculated by normalizing the kinetic current density at 0.9 V to the electrochemical surface area:

$$SA = \frac{MA}{ECSA}$$

SA is reported in mA·cm_{Pt}^{-2}.

MEA fabrication. The as-synthesized catalysts were incorporated into the membrane electrode assembly (MEA) using the catalyst coated membrane (CCM) method, where the catalyst ink was sprayed directly onto a proton exchange membrane (Gore® 8 μm, USA) using a spray machine (Sono-tek®, USA). For the anode catalyst layer, Pt loading is 0.1 $mg_{Pt}\cdot cm^{-2}$ with 30 wt.% Pt-XC72 (JP30S®, China). The catalyst ink was prepared through mixing catalyst and ionomer (25 wt.%, Aquivion® D-79-25BS) into the solvent of n-propanol and DI water with 1:6 volume ratio, where I/C ratio (ionomer/carbon weight ratio) is 0.45. For Heavy-duty Vehicle (HDV) application, the cathode catalyst layer Pt loading was 0.2 $mg_{Pt}\cdot cm^{-2}$ with I/C ratio of 0.55, and all other conditions were the same with anode catalyst layer. The active geometric area of the MEA was 5 cm^2 (3.51 cm × 1.42 cm). Non-woven carbon paper was used as gas diffusion layer (GDL, 22BB Sigracet®, Germany) for both anode and cathode. The MEA is placed between two graphite plates in a sandwich-structure. A differential cell, containing 14 parallel flow channels was employed in our tests, which effectively minimizes the pressure drop from inlet to outlet and enables uniform backpressure across the electrode even with high flow rates.

MEA testing protocols. The MEA was tested on a fuel cell testing station (Fuel Cell Technologies Inc., USA). The testing strictly followed the protocols from the US Department of Energy (DOE). The MEA was first activated (break-in) for 16 hours under H_2 (anode) and air (cathode) with flow rate of 200 standard cubic centimeter per minute (sccm) and 400 sccm, respectively, under 150 kPa_{abs}, 80 °C cell temperature, where the scanning voltage is between 0.7 V to 0.35 V, 50 mV per step and 5 min holding at each point. For H_2-air fuel cell testing, the polarization curves were scanned for voltage from 0.35 V to open circuit voltage (OCV) with 50 mV/step and held for 60 s for each point with anodic (H_2) and cathodic (air) flow rates of 500 sccm and 2000 sccm, respectively, at 80 °C cell temperature and 100% relative humidity (RH), pressure of 250 kPa_{abs} for both sides. The corresponding current density was taken by averaging the values in the last 15 seconds. Catalyst mass activity (MA) was measured at 80 °C and 100% RH under a pressure of 150 kPa_{abs} with anodic (H_2) and cathodic (O_2) flow rates of 500 sccm and 2000 sccm, respectively. The MA was obtained by holding the cell voltage at 0.6 V for 5 min, then holding voltage at 0.9 $V_{iR\text{-}Free}$ for 15 min and taking the average value of current in the last 5 min, which was recorded every second. The MA calculations were corrected with the measured H_2-crossover. MEA stability was evaluated using accelerate stability test (AST) protocol, which was conducted using the trapezoidal wave method from 0.6 V to 0.95 V with 0.5 s rise time and 2.5 s hold time (100 kPa_{abs}, H_2/N_2, 80 °C, 100% RH, 50/75 sccm). The fuel cell performance was evaluated every 30,000 cycles.

Table S1. Separation of ligand effect

Pt-Pt strain applied (%)	Composition beneath 3 layers of Pt(111)	ΔE_{*OH} (eV)
0.00	Pt	0.00
0.00	Pt_5Co	0.01
0.00	Pt_3Co	0.01
0.00	Pt_1Co	0.00

Table S2. *d*-Spacing measured from STEM

	Pt (111)	Pt (110)	Pt (100)
Pt_5Co	0.232	0.277	0.391
Pt_3Co	0.224	0.272	0.386
PtCo	0.212	0.232	0.354

Table S3. Comparison of mass activity (MA) at 0.9 V and MA loss of Pt_5(Ce)Co@Pt catalyst in 0.1 M $HClO_4$ in this work with recently reported efficient Pt-based electrocatalysts in literatures.

**Catalysts**	MA @0.9 V ($A \cdot mg_{Pt}^{-1}$)	SA ($mA \cdot cm^{-2}$)	MA loss	SA loss	AST cycles	Reference
**Pt_5(Ce)Co@Pt**	**2.55**	**3.49**	**31%**	**33%**	**30,000**	**This work**
N-Pt/HEA/C	1.34	1.93	20.9%	16.6%	30,000	Ref[14]
N-HEI/KB	1.45	2.37	17.9%	-	30,000	Ref[15]
PtCo/KB-NH_2	1.82	3.16	13%	-	30,000	Ref[16]
i-CoPt@Pt/KB	2.07	3.95	-	-	30,000	Ref[17]
Pt_3Co/FeN_4	1.34	3.98	~>50%*	-	30,000	Ref[18]
PtCo30Ni10@NG	1.36	~1.85	18.2%	13.5%	10,000	Ref[19]
Pt3Co0.6Ti0.4/DMC	1.49	0.22	20.1%	22.7%	20,000	Ref[20]

PtCo/Sn–N–C	1.65	2.66	21.8%	-	30,000	Ref[21]
PtCo-H/P	2.21	4.0	34%	25%	30,000	Ref[22]
PtCo/$P_{2.73}O_x$–KB	1.11	1.79	24.3%	~33.3%	50,000	Ref[23]
PtCo/Co–N–C	0.70	-	44.43%	-	30,000	Ref[24]

*Estimated data based on polarization curves.

Table S4. Summary of fuel cell performance of the recently reported Pt-based intermetallic electrocatalysts.

**Catalysts**	**Cathode loading (mg_{Pt} cm^{-2})**	**Outlet pressure (kPa_{abs})**	**H_2/Air flow rate (sccm)**	**Current density ($A\ cm^{-2}$)**		**Mass activity @0.9 $V_{iR\text{-}free}$ ($A\ mg_{Pt}^{-1}$)**		**Reference**
				**BOL**	**Loss 30k**	**BOL**	**Loss 30k**	
**Pt_5(Ce)Co@Pt**	**0.18**	**250**	**500/2000**	**1.88 (@0.7 V)**	**15%**	**0.78**	**44%**	**This work**
	**0.09**	**150**	**55/2000**	**0.54 (@0.8 V)**	**27%**	**1.58**	**54%**	
i-CoPt@Pt/KB	0.1	250	200/800	1.02 (@0.67 V)	-	0.53	34%	Ref[17]
Pt_3Co/FeN_4	0.1	150	500/1000	1.23 (@0.67 V)	~19%	0.72	38%	Ref[18]
N-Pt/HEA/C	0.2	250	500/2000	1.64 (@0.7 V)	12.3%	0.26	7.9%	Ref[14]
Pt3Co0.6Ti0.4/DMC	0.2	250	-	1.35 (@0.6 V)	22.9%	-	-	Ref[20]
$PtCu_{0.72}Co_{0.01}$	0.2	200	300/150	1.55 (@0.6 V)	-	-	-	Ref[25]
$L1_2$-Pt_3Co/NC-Cys	0.25	250	500/2000	1.44 (@0.7 V)	-	0.56	-	Ref[26]
PtCo/Sn–N–C	0.1	150	1000/1000	~1.2 (@0.7 V)	-	0.94	2.1%	Ref[21]
N-HEI/KB	0.2	250	500/2000	1.52 (@0.7 V)	8.5%	-	-	Ref[15]

Table S5. Current density at 0.7 V decay rates for Pt_5(Ce)Co@Pt, Pt_3Co@Pt, PtCo@Pt and Pt/C under HDV condition in MEA testing

Catalysts	Interval	Δj ($A\cdot cm^{-2}$ per 30,000 cycles)	Δj ($A\cdot cm^{-2}$ per 10,000 cycles)	% decay per 30,000 cycles	% decay per 10,000 cycles
Pt_5(Ce)Co@Pt	0–30k	−0.29	−0.097	−15.4%	−5.1%
	30–60k	−0.09	−0.030	−5.7%	−1.9%
	60–90k	−0.08	−0.027	−5.3%	−1.8%
	90–120k	−0.09	−0.030	−6.3%	−2.1%
	120–150k	−0.08	−0.027	−6.0%	−2.0%
	150–180k	−0.08	−0.027	−6.4%	−2.1%
Pt_3Co@Pt	0–30k	−0.23	−0.076	−4.6%	−13.9%
PtCo@Pt	0–30k	−0.30	−0.010	−7.1%	−21.3%
Commercial Pt/C	0–30k	−0.22	−0.073	−7.6%	−22.9%

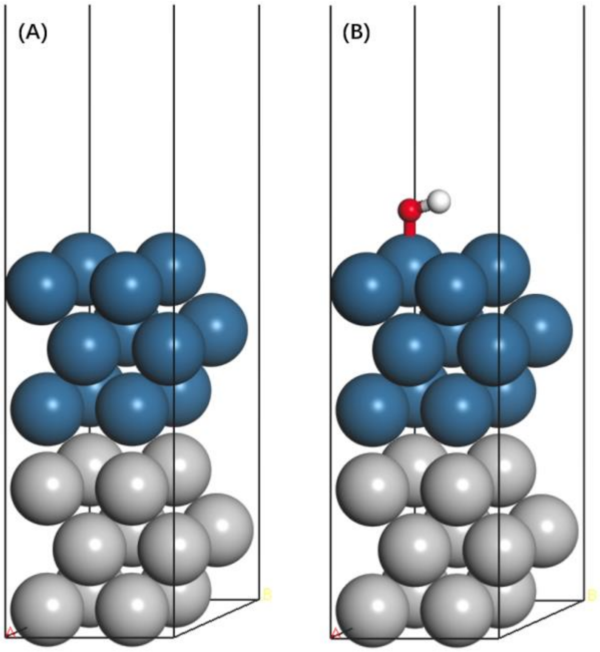

Figure S1. Slab cell of base@Pt (A) clean surface (B) surface with *OH (Blue: Pt(111) shell relaxed; Grey: core material constrained)

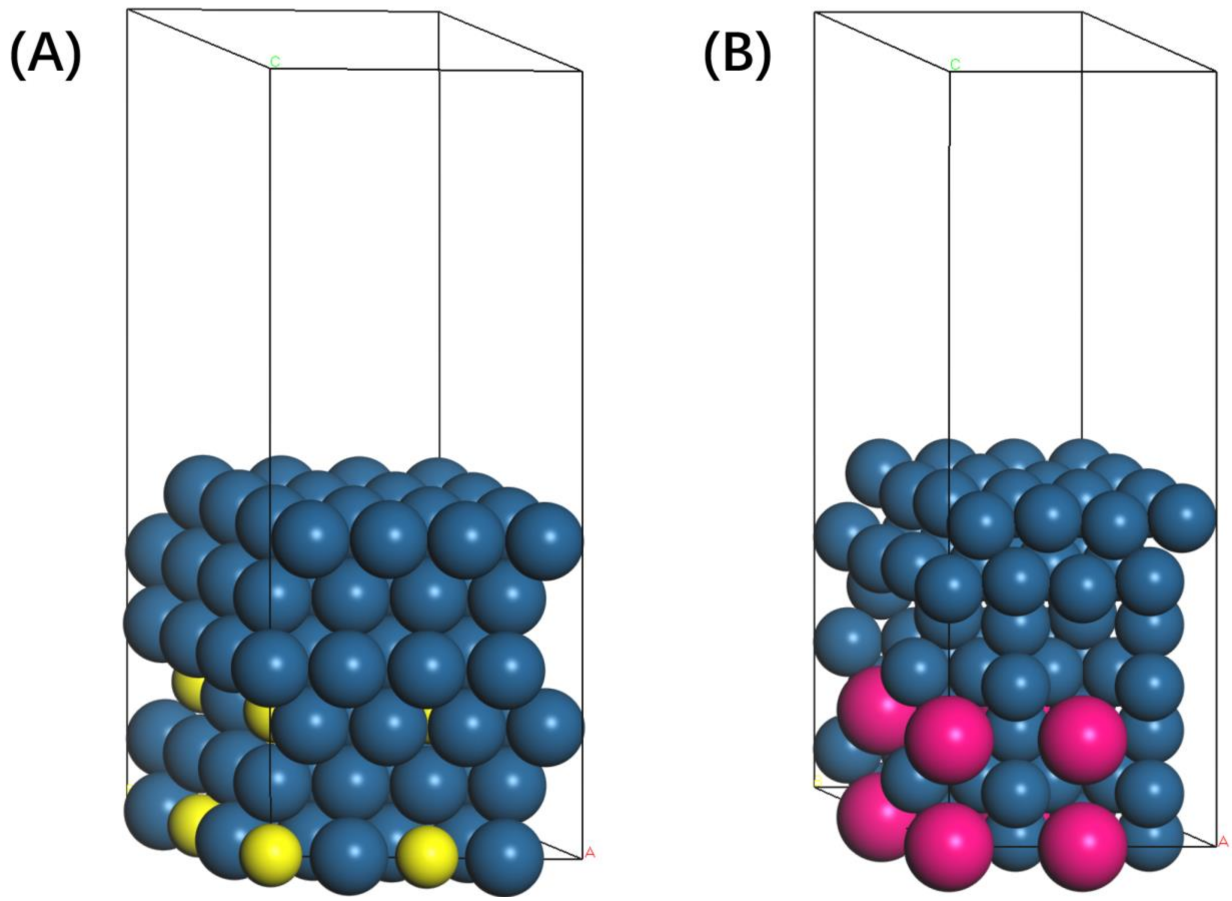

Figure S2. The slab model of (A) Pt_5Co@Pt that 3 layers of Pt(111) is supported on Pt_5Co(111) base (B) Pt_5Ce@Co that 3 layers of Pt(111) is supported on Pt_5Ce(0001) base with a *Kagomé* interlayer (Blue: Pt, Yellow: Co, Pink: Ce)

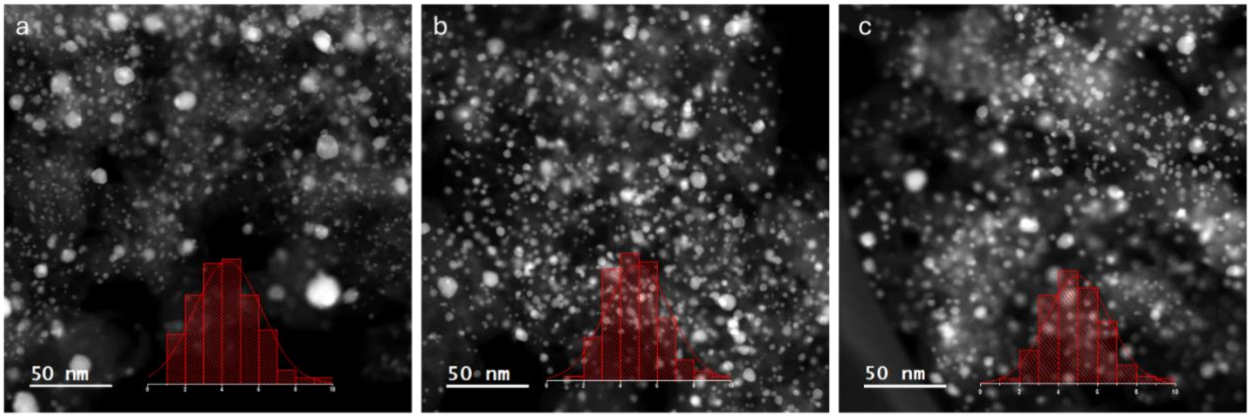

Figure S3. Particle size distributions shown in HAAADF-STEM images of (**a**) $Pt_5(Ce)Co$, (**b**) Pt_3Co, (**c**) PtCo.

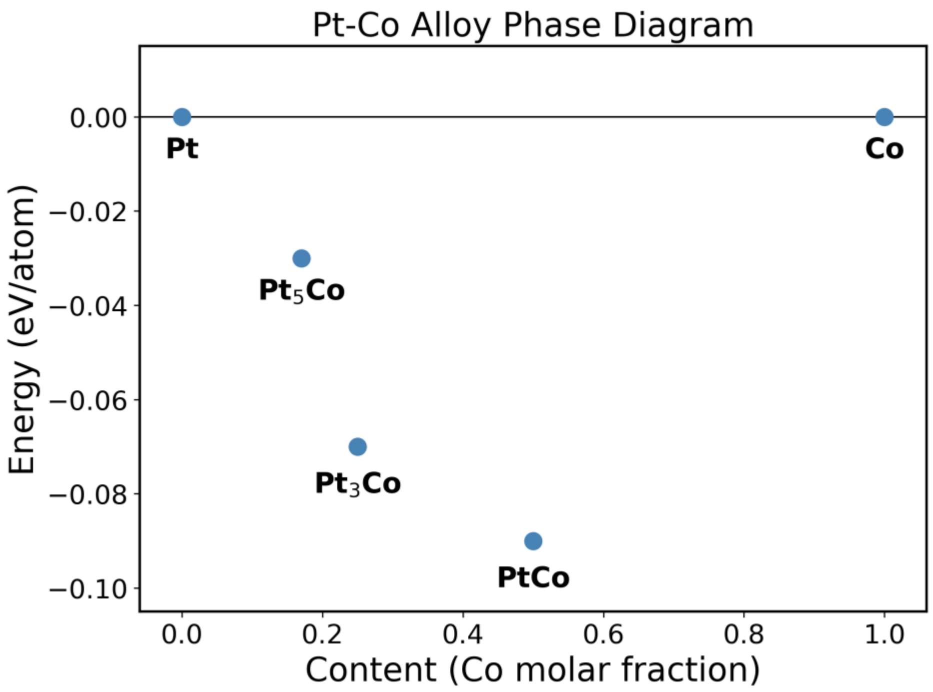

Figure S4. Pt-Co phase diagram

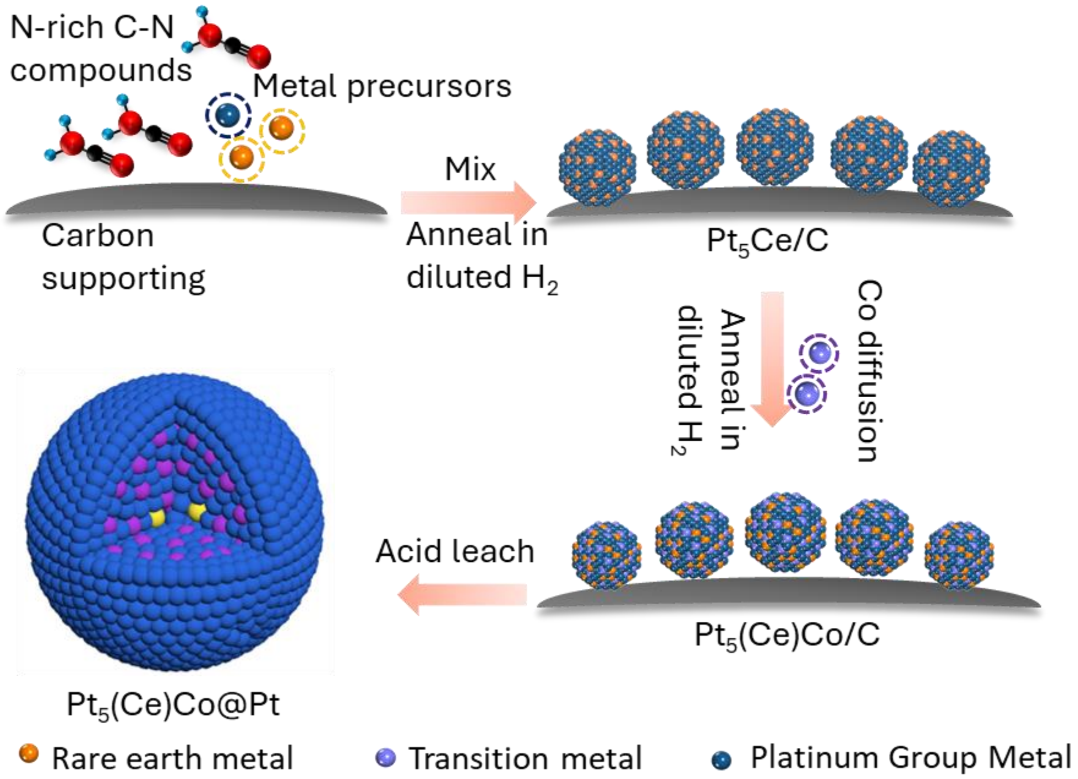

Figure S5. Synthesis process schematic

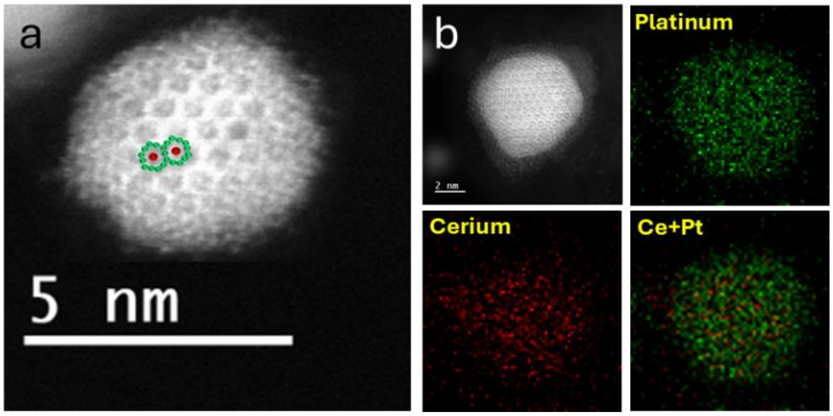

Figure S6. a) HAADF-TEM and **b**) EDS mapping of binary precursor Pt_5Ce/C

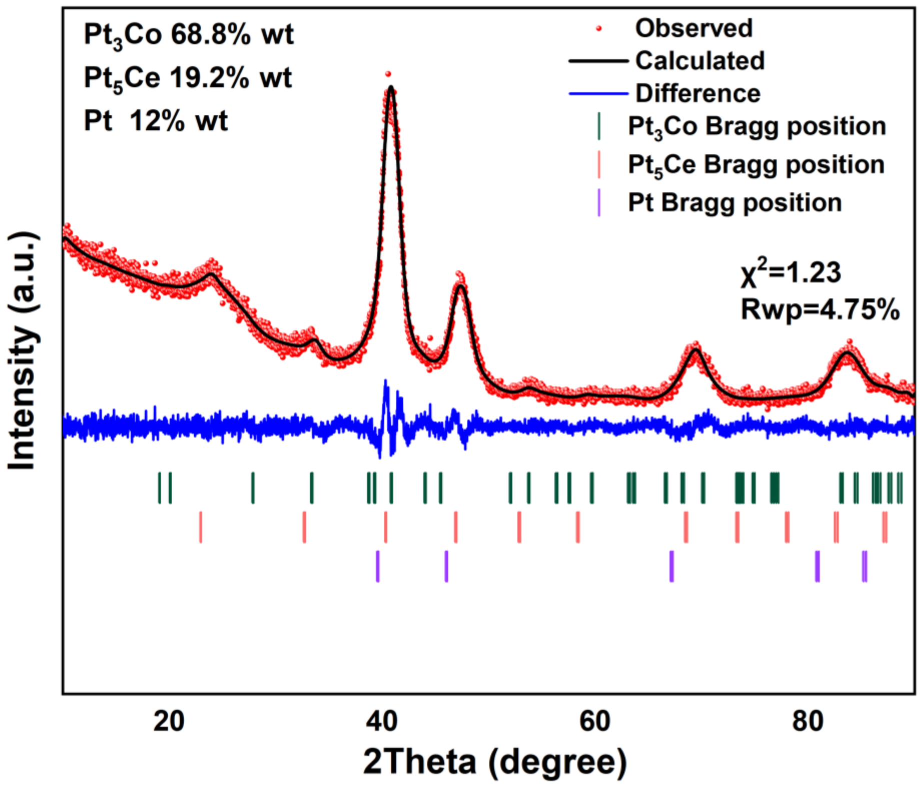

Figure S7. Rietveld refinement profile of XRD data of as prepared $Pt_5(Ce)Co@Pt$. The dots correspond to the experimental data and the solid line is for the Rietveld refinement fit. The lower blue curve is the difference between the observed and calculated at each step.

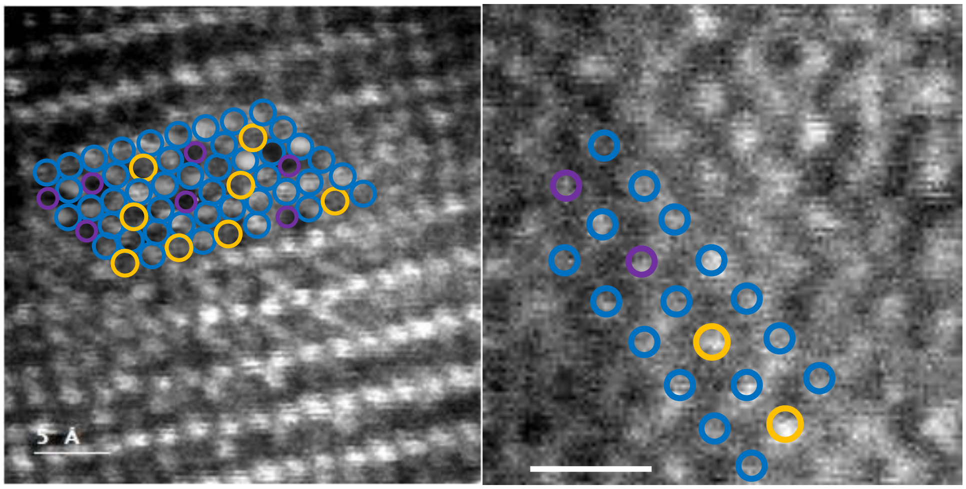

Figure S8. Enlarged HAADF-STEM image from **Figure 2b** core region, blue circles represent Pt atoms, yellow circles represent Ce atoms, and purple circles represent Co atoms.

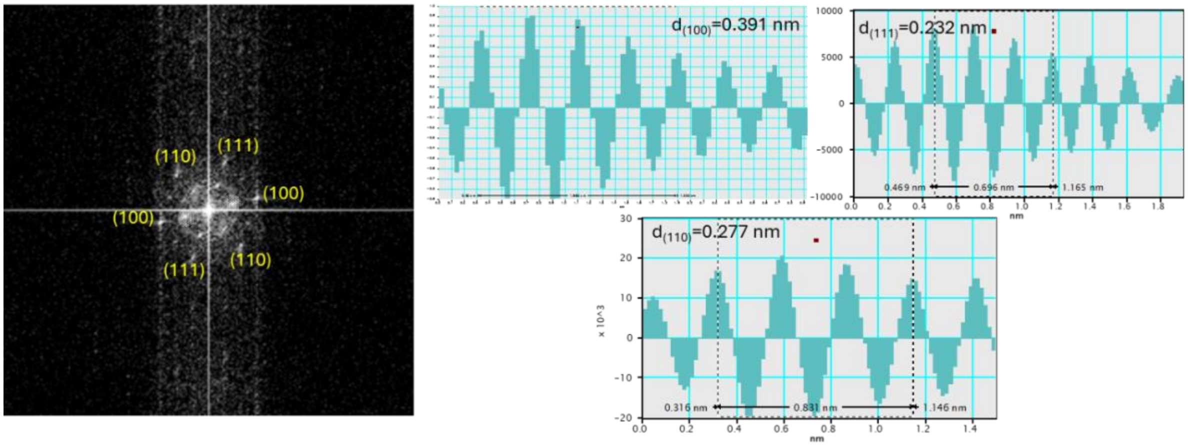

Figure S9. TEM IFFT profiles of $Pt_5(Ce)Co$

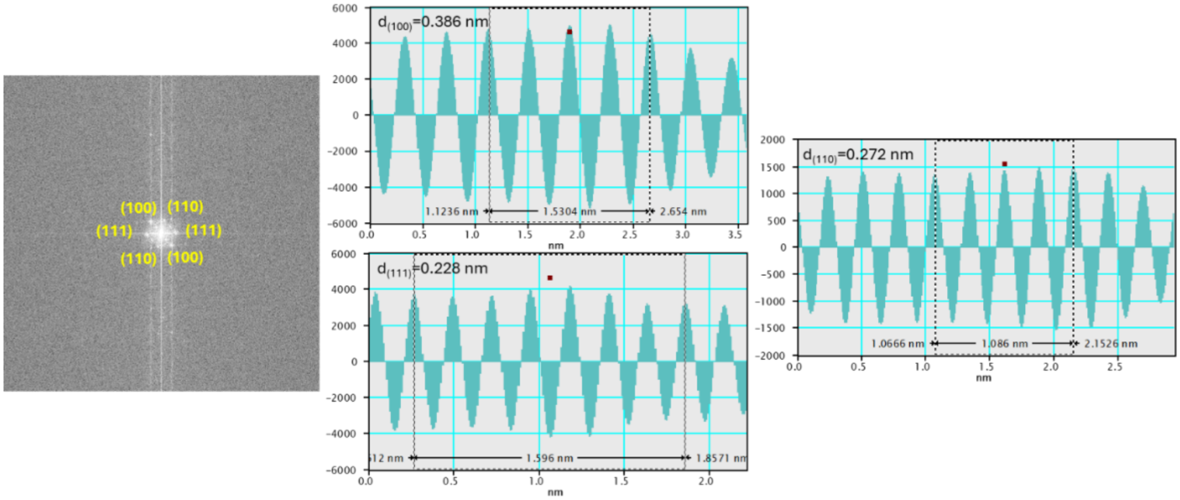

Figure S10. TEM IFFT profiles of Pt_3Co.

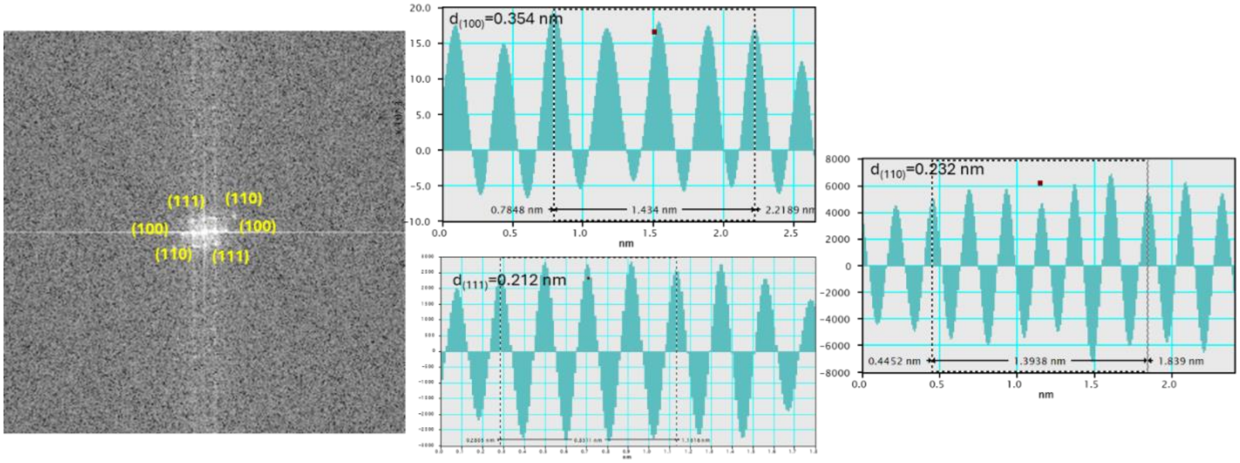

Figure S11. TEM IFFT profiles of PtCo.

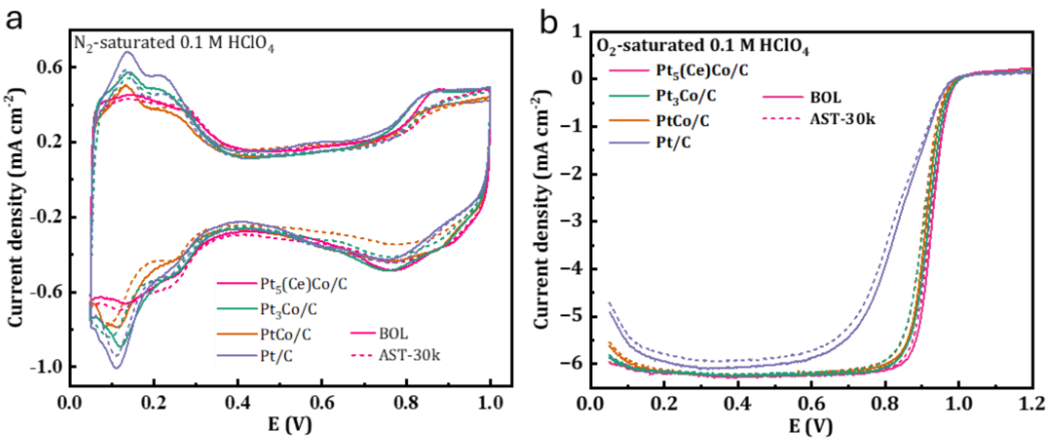

Figure S12. a) CV curves and b) ORR polarization curves before and after 30,000 cycles of $Pt_5(Ce)Co@Pt$, $Pt_3Co@Pt$, PtCo@Pt and Pt/C in RDE testing.

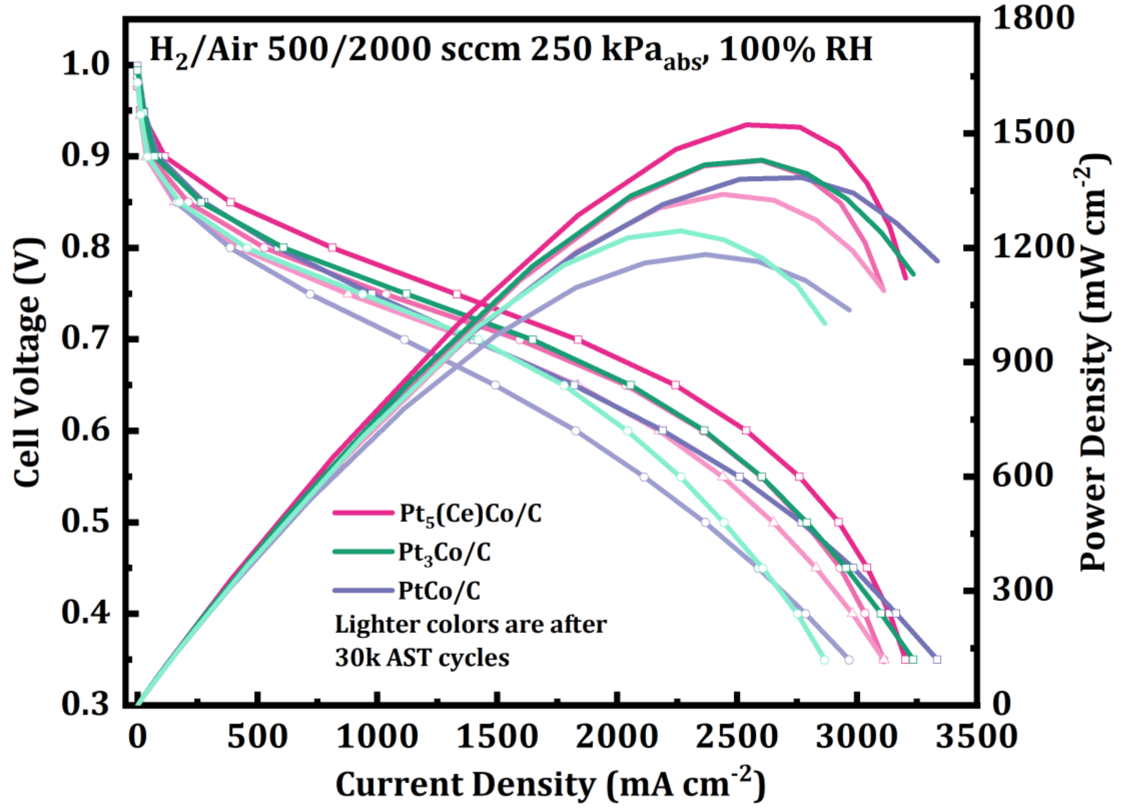

Figure S13. H_2/air polarization curves and power density curves before and after 30,000 cycles of $Pt_5(Ce)Co@Pt$, $Pt_3Co@Pt$, PtCo@Pt and commercial Pt/C in MEA testing. Anode: 30 wt.% JP30S 0.1 mg_{Pt}/cm^2; Cathode: CATALYST, 0.18 mg_{Pt}/cm^2; Test conditions: Differential Cell, 500/2000 sccm, H_2/Air 250 kPa_{abs}, Operation temperature 80 °C, and 100% relative humidity.

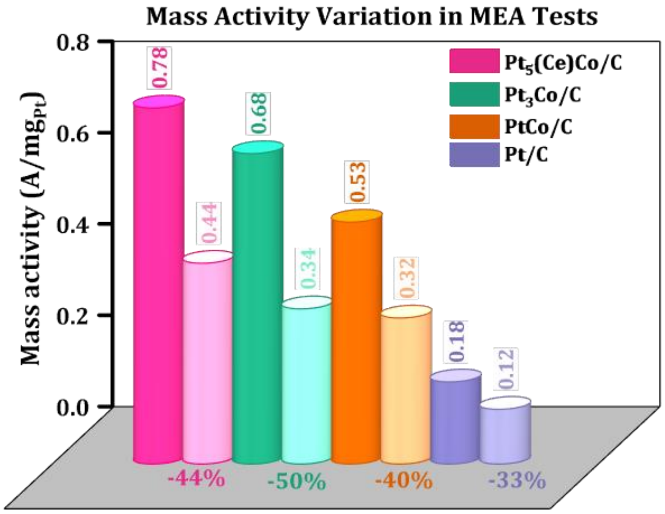

Figure S14. Mass activity loss after 30,000 AST cycles in MEA testing under HDV, where mass activity was tested under 150 kPa_{abs}.

References

(1) Kresse, G.; Joubert, D. From ultrasoft pseudopotentials to the projector augmented-wave method. *Physical Review B* **1999**, *59* (3), 1758-1775. DOI: 10.1103/PhysRevB.59.1758.

(2) Kresse, G.; Furthmüller, J. Efficiency of ab-initio total energy calculations for metals and semiconductors using a plane-wave basis set. *Comput. Mater. Sci.* **1996**, *6* (1), 15-50. DOI: 10.1016/0927-0256(96)00008-0.

(3) Kresse, G.; Furthmüller, J. Efficient iterative schemes for ab initio total-energy calculations using a plane-wave basis set. *Phys. Rev. B Condens. Matter* **1996**, *54* (16), 11169-11186. DOI: 10.1103/physrevb.54.11169.

(4) Perdew, J. P.; Burke, K.; Ernzerhof, M. Generalized Gradient Approximation Made Simple. *Phys. Rev. Lett.* **1996**, *77* (18), 3865-3868. DOI: 10.1103/PhysRevLett.77.3865.

(5) Press, W. H.; Flannery, B. P.; Teukolsky, S. A.; Vetterling, W. T. *Numerical Recipes: The Art of Scientific Computing*; Cambridge University Press, 1986.

(6) Pulay, P. Convergence Acceleration of Iterative Sequences. The Case of SCF Iteration. *Chem. Phys. Lett.* **1980**, *73*, 393-398.

(7) III, R. D. J. NIST Computational Chemistry Comparison and Benchmark DataBase. In *NIST Standard Reference Database Number 101*.

(8) Chase, M. W., Jr. *NIST-JANAF Thermochemical Tables*; 1998. DOI: 10.18434/t42s31.

(9) *Standard Potentials in Aqueous Solutions*; Dekker, 1985.

(10) Milazzo, G.; Caroli, S.; Sharma, V. K. *Tables of Standard Electrode Potentials*; Wiley, 1972.

(11) Swift, E. H.; Butler, E. A. *Quantitative Measurements and Chemical Equilibria*; Freeman, 1972.

(12) Tripkovic, V.; Zheng, J.; Rizzi, G. A.; Marega, C.; Durante, C.; Rossmeisl, J.; Granozzi, G. Comparison between the Oxygen Reduction Reaction Activity of Pd5Ce and Pt5Ce: The Importance of Crystal Structure. *ACS Catalysis* **2015**, *5* (10), 6032-6040. DOI: 10.1021/acscatal.5b01254.

(13) Kulkarni, A.; Siahrostami, S.; Patel, A.; Norskov, J. K. Understanding Catalytic Activity Trends in the Oxygen Reduction Reaction. *Chem Rev* **2018**, *118* (5), 2302-2312. DOI: 10.1021/acs.chemrev.7b00488 From NLM PubMed-not-MEDLINE.

(14) Zhao, X.; Cheng, H.; Chen, X.; Zhang, Q.; Li, C.; Xie, J.; Marinkovic, N.; Ma, L.; Zheng, J. C.; Sasaki, K. Multiple Metal-Nitrogen Bonds Synergistically Boosting the Activity and Durability of High-Entropy Alloy Electrocatalysts. *J Am Chem Soc* **2024**, *146* (5), 3010-3022. DOI: 10.1021/jacs.3c08177 From NLM PubMed-not-MEDLINE.

(15) Zhao, X.; Cheng, H.; Wu, L.; Zhang, Q.; Chen, X.; Marinkovic, N.; Li, C.; Tan, S.; Hu, E.; Ma, L.; et al. Sub-angstrom strain in high-entropy intermetallic boosts the oxygen reduction reaction in fuel cell cathodes. *Nat Commun* **2025**, *16* (1), 7547. DOI: 10.1038/s41467-025-62725-7 From NLM PubMed-not-MEDLINE.

(16) Gong, Q.; Zhang, H.; Yu, H.; Jeon, S.; Ren, Y.; Yang, Z.; Sun, C.-J.; Stach, E. A.; Foucher, A. C.; Yu, Y.; et al. Amino-tethering synthesis strategy toward highly accessible sub-3-nm L10-PtM catalysts for high-power fuel cells. *Matter* **2023**, *6* (3), 963-982. DOI: 10.1016/j.matt.2022.12.011.

(17) Yoo, T. Y.; Lee, J.; Kim, S.; Her, M.; Kim, S.-Y.; Lee, Y.-H.; Shin, H.; Jeong, H.; Sinha, A. K.; Cho, S.-P.; et al. Scalable production of an intermetallic Pt–Co electrocatalyst for high-power

proton-exchange-membrane fuel cells. *Energy & Environmental Science* **2023**, *16* (3), 1146-1154. DOI: 10.1039/d2ee04211h.
(18) Qiao, Z.; Wang, C.; Li, C.; Zeng, Y.; Hwang, S.; Li, B.; Karakalos, S.; Park, J.; Kropf, A. J.; Wegener, E. C.; et al. Atomically dispersed single iron sites for promoting Pt and Pt3Co fuel cell catalysts: performance and durability improvements. *Energy & Environmental Science* **2021**, *14* (9), 4948-4960. DOI: 10.1039/d1ee01675j.
(19) Sun, L.; Yin, Y.; Ren, B.; Qin, Y.; Wen, G.; Chen, Z. ZIF-derived ternary Pt-Co-Ni alloy as the superior active and durable catalyst for PEMFC. *Nano Energy* **2024**, *120*. DOI: 10.1016/j.nanoen.2023.109154.
(20) Zhao, W.; Chi, B.; Liang, L.; Yang, P.; Zhang, W.; Ge, X.; Wang, L.; Cui, Z.; Liao, S. Optimizing the Electronic Structure of Ordered Pt–Co–Ti Ternary Intermetallic Catalyst to Boost Acidic Oxygen Reduction. *ACS Catalysis* **2022**, *12* (13), 7571-7578. DOI: 10.1021/acscatal.2c00554.
(21) Huang, Z.; Xiao, Q.; Ding, T.; Xia, J.; Zhan, C.; Meng, X.; Pao, C.-W.; Hu, Z.; Huang, W.-H.; Wang, Y.; et al. Interfacial metal–coordinated bifunctional PtCo for practical fuel cells. *Science Advances* **2025**, *11* (10), eadt4914. DOI: 10.1126/sciadv.adt4914 (acccessed 2025/07/24).
(22) Zeng, W.-J.; Wang, C.; Yin, P.; Tong, L.; Yan, Q.-Q.; Chen, M.-X.; Xu, S.-L.; Liang, H.-W. Alloying Matters for Ordering: Synthesis of Highly Ordered PtCo Intermetallic Catalysts for Fuel Cells. *Inorganic Chemistry* **2023**, *62* (13), 5262-5269. DOI: 10.1021/acs.inorgchem.3c00331.
(23) Hu, S.-N.; Xu, W.-C.; Tian, N.; Chen, S.-M.; Li, M.-Y.; Shen, J.-F.; Lin, J.-X.; Guo, S.-L.; Huang, X.-Y.; Zhou, Z.-Y.; et al. A P–O functional group anchoring Pt–Co electrocatalyst for high-durability PEMFCs. *Energy & Environmental Science* **2024**, *17* (9), 3099-3111. DOI: 10.1039/d3ee04503j.
(24) Guo, P.; Xia, Y.; Liu, B.; Ma, M.; Shen, L.; Dai, Y.; Zhang, Z.; Zhao, Z.; Zhang, Y.; Zhao, L.; et al. Low-Loading Sub-3 nm PtCo Nanoparticles Supported on Co–N–C with Dual Effect for Oxygen Reduction Reaction in Proton Exchange Membrane Fuel Cells. *ACS Applied Materials & Interfaces* **2022**, *14* (48), 53819-53827. DOI: 10.1021/acsami.2c15996.
(25) Hu, B.; Yuan, J.; Zhang, J.; Shu, Q.; Guan, D.; Yang, G.; Zhou, W.; Shao, Z. High activity and durability of a Pt–Cu–Co ternary alloy electrocatalyst and its large-scale preparation for practical proton exchange membrane fuel cells. *Composites Part B: Engineering* **2021**, *222*, 109082. DOI: https://doi.org/10.1016/j.compositesb.2021.109082.
(26) Liang, J.; Yu, H.; Zachman, M. J.; Hwang, S.; Qi, M.; Zeng, Y.; Zhang, B.; Li, J.; Guo, J.; Dun, C.; et al. Creating Favorable Pt/Co Interfaces via a Two-Step Approach for Constructing Highly Durable PtCo Intermetallic Fuel Cell Catalysts. *Adv Mater* **2025**, e10847. DOI: 10.1002/adma.202510847 From NLM Publisher.
